# Polysialic Acid Is Required for Dopamine D2 Receptor-Mediated Plasticity Involving Inhibitory Circuits of the Rat Medial Prefrontal Cortex

**DOI:** 10.1371/journal.pone.0029516

**Published:** 2011-12-28

**Authors:** Esther Castillo-Gómez, Emilio Varea, José Miguel Blasco-Ibáñez, Carlos Crespo, Juan Nacher

**Affiliations:** 1 Neurobiology Unit and Program in Basic and Applied Neurosciences, Cell Biology Department, Universitat de València, Burjassot, Valencia, Spain; 2 Fundación Investigación Hospital Clínico de Valencia, INCLIVA, Valencia, Spain; Chiba University Center for Forensic Mental Health, Japan

## Abstract

Decreased expression of dopamine D2 receptors (D2R), dysfunction of inhibitory neurotransmission and impairments in the structure and connectivity of neurons in the medial prefrontal cortex (mPFC) are involved in the pathogenesis of schizophrenia and major depression, but the relationship between these changes remains unclear. The polysialylated form of the neural cell adhesion molecule (PSA-NCAM), a plasticity-related molecule, may serve as a link. This molecule is expressed in cortical interneurons and dopamine, via D2R, modulates its expression in parallel to that of proteins related to synapses and inhibitory neurotransmission, suggesting that D2R-targeted antipsychotics/antidepressants may act by affecting the plasticity of mPFC inhibitory circuits. To understand the role of PSA-NCAM in this plasticity, rats were chronically treated with a D2R agonist (PPHT) after cortical PSA depletion. PPHT-induced increases in GAD67 and synaptophysin (SYN) neuropil expression were blocked when PSA was previously removed, indicating a role for PSA-NCAM in this plasticity. The number of PSA-NCAM expressing interneuron somata also increased after PPHT treatment, but the percentages of these cells belonging to different interneuronal subpopulations did not change. Cortical pyramidal neurons did not express PSA-NCAM, but puncta co-expressing this molecule and parvalbumin could be found surrounding their somata. PPHT treatment increased the number of PSA-NCAM and parvalbumin expressing perisomatic puncta, but decreased the percentage of parvalbumin puncta that co-expressed SYN. PSA depletion did not block these effects on the perisomatic region, but increased further the number of parvalbumin expressing puncta and increased the percentage of puncta co-expressing SYN and parvalbumin, suggesting that the polysialylation of NCAM may regulate perisomatic inhibition of mPFC principal neurons. Summarizing, the present results indicate that dopamine acting on D2R influences structural plasticity of mPFC interneurons and point to PSA-NCAM as a key player in this remodeling.

## Introduction

During recent years, several evidences indicate that, in addition to neurochemical alterations, changes in the structure and connectivity of neurons in the medial prefrontal cortex (mPFC) may also underlie the pathogenesis of different psychiatric disorders, including schizophrenia and major depression [1]–[2]. Dopamine and serotonin play a crucial role in the regulation of mPFC function and in the etiology and treatment of these disorders. Moreover, there is evidence indicating that changes in monoamine neurotransmission can induce neuronal structural remodeling in the adult CNS. Although most of the studies on neuronal structural plasticity have been focused on principal neurons, there is abundant evidence that, in these psychiatric disorders, interneurons and cortical inhibitory networks show abnormalities [3]–[5]. The polysialylated form of the neural cell adhesion molecule (PSA-NCAM) is a good candidate to mediate these structural changes, especially in interneurons. The addition of long chains of polysialic acid to NCAM confers anti-adhesive properties, which favor plastic processes such as neuronal migration and the growth or remodeling of neurites, spines and synapses [6]–[8]. In rodents, the expression of PSA-NCAM is very high during embryonic and early postnatal development but subsequentially decreases. However, expression of this molecule can be still observed in the adult mPFC and persists stable until old age [9]–[10]. In this cortical region, PSA-NCAM is exclusively expressed by a subset of interneurons and it is found in their somata and in inhibitory elements in the neuropil [9]. These interneurons have reduced dendritic arborization, spine density and less synaptic input than interneurons lacking PSA-NCAM [11]. Our laboratory has described that manipulation of dopaminergic or serotoninergic neurotransmission in the mPFC of adult rats leads to changes in the expression of PSA-NCAM [12]–[13]. Dopamine-induced changes may probably take place through the action of D2 receptors (D2R), which are expressed in PSA-NCAM expressing interneurons of the mPFC, since D2R agonists, such as 2-(N-Phenethyl-N-propyl) amino-5-hydroxytetralin hydrochloride (PPHT), increased PSA-NCAM expression and D2R antagonists (haloperidol) decreased it. Interestingly, this modulation of PSA-NCAM expression occurs in parallel to changes in the expression of synaptic proteins and molecules related to inhibitory neurotransmission, which strongly suggests the involvement of PSA-NCAM in the plasticity of prefrontocortical inhibitory circuits [12].

In order to better understand the influence of dopamine through D2R and PSA-NCAM in the structural plasticity of the mPFC, we have replicated our experiment using PPHT [12] in rats in which PSA had been previously depleted from the mPFC using the specific enzyme Endo-N-acetylneuraminidase (Endo-N). We have studied whether PPHT-induced changes in synaptic proteins and molecules related to inhibitory neurotransmission could be blocked by PSA depletion, as well as the effects of PPHT and Endo-N on the perisomatic innervation of pyramidal neurons in the mPFC. The effects of PPHT on the number and neurochemical phenotype of PSA-NCAM expressing somata in this region have also been studied.

## Materials and Methods

### Ethics statement

All animal experimentation was conducted in accordance with the Directive 2010/63/EU of the European Parliament and of the Council of 22 September 2010 on the protection of animals used for scientific purposes and was approved by the Committee on Bioethics of the Universitat de València (permit number: A1234271133556). Every effort was made to minimize the number of animals used and their suffering.

### Animals

Adult male Sprague-Dawley rats (3 months-old; 296±13 g; Harlan Interfauna Iberica S.L., Barcelona, Spain) were used for all experimental procedures. Twenty-four rats (6 rats in each of the 4 experimental groups) were used to study the effects of D2R agonist chronic treatment after specific polysialic acid (PSA) depletion from the mPFC (see next section) and four rats to analyze the neurochemical phenotype of PSA-NCAM expressing puncta surrounding pyramidal cell somata.

Animals were housed in groups of 3 in a temperature- and humidity-controlled environment and maintained on a 12 h light/dark cycle with food and water available *ad libitum*. Rats were allowed to habituate to our facilities one week prior to the start of the experiments.

### Polysialic acid depletion and D2R agonist chronic treatment

Twenty-four rats (6 rats in each of the 4 experimental groups) were unilaterally injected in the secondary motor cortex with 1 µl of either the enzyme Endo-N (AbCys, Paris, France) or a vehicle solution and, one week later, they were treated for 7 days with either PPHT or saline as described before [12]. Body weight was measured on the day of surgery (day 0) and in days 7, 10 and 14. Please, see **table S1** and **material and methods** for a detailed explanation on the procedures and body weight statistical analysis.

### Histological procedures

Rats were perfused transcardially with 4% paraformaldehyde solution for 30 min. Brains were extracted from the skull and their hemispheres were separated.

Both hemispheres of control rats (n = 4) and the ipsilateral hemispheres respective to the side of injection of experimental rats (n = 24) were cut in coronal sections (50 µm) and then, the slices were collected in 10 sequential subseries.

More details on the histological procedures used can be found in **material and methods S1**.

### Immunohistochemistry for conventional light microscopy

In order to analyze whether Endo-N injection and/or PPHT treatment induced changes on the neuropil expression of PSA-NCAM, SYN, GAD67 and NCAM, four subseries from each animal were processed “free-floating” for immunohistochemistry using the avidin-biotin-peroxidase (ABC) method as described before [9], [12]. The detailed procedure can be found in **material and methods S1**. For further information on the antibodies used, see **table 1**.

**Table 1:** Primary and secondary antibodies.

Primary antibodies (abbreviated names)						
	Host	Isotype	Dilution	Incubation	Company	References
Anti-CB	Rabbit	IgG	1∶2000	O/N, 25°C	Swant	Hédou et al. 2002
Anti-CR	Rabbit	IgG	1∶2000	O/N, 25°C	Swant	Schwaller et al., 1993
Anti-CaMKII-α	Mouse	IgG1	1∶500	36 h, 4°C	Abcam	Benson et al., 1992
Anti-GAD67	Mouse	IgG2a	1∶500	O/N, 25°C	Chemicon-Millipore	Varea et al., 2005
Anti-GAD65/67	Rabbit	IgG	1∶1000	O/N, 25°C	Chemicon-Millipore	Benagiano et al., 2007
Anti-NCAM	Mouse	IgG2b	1∶200	O/N, 25°C	DSHB	dshb.biology.uiowa.edu
Anti-PSA-NCAM	Mouse	IgM	1∶700	36 h, 4°C	Abcys	Theodosis et al, 1991
Anti-PV(1)	Rabbit	IgG	1∶2000	O/N, 25°C	Swant	Miettinen et al., 1996
Anti-PV	Guinea pig	IgG	1∶2000	36 h, 4°C	Synaptic Systems	www.sysy.com
Anti-SYN(2)	Mouse	IgG1	1∶500	O/N, 25°C	Sigma-Aldrich	Devoto and Barnstable, 1989
Anti-SYN	Rabbit	IgG	1∶1000	O/N, 25°C	Chemicon-Millipore	Hanaya et al., 2007
Anti-VGAT	Rabbit	IgG	1∶1000	O/N, 25°C	Synaptic Systems	Takamori et al.,2000
Anti-VGLUT1	Guinea pig	IgG	1∶2000	O/N, 25°C	Chemicon-Millipore	Melone et al., 2005

Secondary antibodies						
	Host	Label	Dilution	Incubation	Company	
Anti-Guinea pig IgG	Donkey	Cy™5	1∶400	1 h, 25°C	Jackson immunoResearch	
Anti-Mouse IgM	Donkey	Biotin-SP	1∶400	1 h, 25°C	Jackson immunoResearch	
Anti-Mouse IgM	Goat	Alexa Fluor® 555	1∶400	1 h, 25°C	Molecular Probes	
Anti-Mouse IgG	Donkey	Biotin-SP	1∶400	1 h, 25°C	Jackson immunoResearch	
Anti-Mouse IgG1(3)	Goat	Biotin-SP	1∶400	1 h, 25°C	Jackson immunoResearch	
Anti-Rabbit IgG	Donkey	Alexa Fluor® 488	1∶400	1 h, 25°C	Molecular Probes	
Anti-Rabbit IgG	Donkey	Alexa Fluor® 647	1∶400	1 h, 25°C	Molecular Probes	

(1) Used in CaMKIIα - PSA-NCAM - PV triple immunohistochemistry.

(2) Used in SYN immunohistochemistry for light microscopy.

(3) Used in multiple-labeling experiments for the detection of anti-CaMKII-α primary antibody when other primary antibody generated in mouse was also being used. The Avidin, NeutrAvidin®, Texas Red® conjugate (1∶200, Molecular Probes) or streptavidin, Alexa Fluor ® 488 conjugate were used before to detect biotin.

Abbreviations: CB, calbindin-D28k; CR , calretinin; CaMKII-α, α subunit of the Ca^2+^/calmodulin dependent protein kinase II; GAD67, 67 kDa isoform of the glutamate decarboxilase enzime, GAD65/67, both 65 and 67 kDa isoforms of the glutamate decarboxilase enzyme; PSA-NCAM, polysialylated form of de neural cell adhesion molecule; PV, parvalbumin; SYN, synaptophysin; VGAT, vesicular γ-aminobutyric acid (GABA) transporter; VGLUT1, vesicular glutamate transporter 1.

PSA-NCAM immunohistochemistry was also used to analyze the number of PSA-NCAM expressing interneurons after PPHT treatment and to validate the effectiveness of Endo-N treatment.

### Quantification of neuropil immunoreactivity and estimation of the total number of neurons expressing PSA-NCAM, CB, CR or PV

In animals treated with Endo-N and/or PPHT, the intensity of PSA-NCAM, SYN, GAD67 and NCAM immunoreactivities in the mPFC neuropil were determined using a previously described methodology [9], [12]–[14].

The number of PSA-NCAM expressing interneurons in the different layers and regions of mPFC was estimated using a modified version of the fractionator method [15], as described before [13], [16].

See **material and methods S1** and **figure S3** for further details on methodology and statistical analysis.

### Immunohistochemistry for confocal microscopy

Fifty µm-thick coronal sections were processed “free-floating” for immunohistochemistry as described before [11], [12]. See **table 1** and **material and methods S1** for further details on antibodies, specificity and immunohistochemical procedures.

In order to analyze the neurochemical phenotype of PSA-NCAM expressing puncta surrounding pyramidal neuron somata in control animals, we performed seven different triple immunostainings: anti-PSA-NCAM and anti-α subunit of the Ca^2+^/calmodulin dependent protein kinase II (CaMKII-α) primary antibodies in combination with (1) anti-GAD65/67, (2) anti-vesicular GABA transporter (VGAT), (3) anti-VGLUT1, 4) anti-SYN, 5) anti-parvalbumin (PV), 6) anti-calretinin (CR) or (7) anti-calbindin (CB) antibodies.

In sections obtained from experimental rats, we performed a triple immunostaining against PSA-NCAM, PV and CB, and a double immunostaining against PSA-NCAM and CR, to analyze the neurochemical phenotype of PSA-NCAM expressing interneurons after PPHT treatment and to estimate the number of interneurons expressing CB, CR or PV in the different regions of the mPFC (using a modified version of the fractionator method) [13], [15], [16].

In order to study the perisomatic innervation of mPFC pyramidal neurons after treatment, we performed two double and a triple immunostaining using anti-CaMKII-α primary antibody in combination with either (1) anti-PSA-NCAM, (2) anti-GAD65/67 or (3) anti-PV and anti-SYN primary antibodies.

### Analysis of the neurochemical phenotype of PSA-NCAM expressing interneurons after PPHT treatment

Sections double or triple labeled for PSA-NCAM and interneuronal subpopulation markers (PV, CB, CR) were observed under a confocal microscope (Leica TCS-SPE) using a 63× oil objective. Z-series of optical sections (1 µm apart) were obtained using sequential scanning mode and stacks were then processed with Zeiss LSM 5 image software. Fifty PSA-NCAM immunoreactive neurons within the mPFC were randomly selected from each animal and immunostained to determine the co-expression of PSA-NCAM and each marker. Percentages of co-localization were determined for each animal and means ± S.E.M. were calculated. The resulting values (Control/PPHT vs Control/Control) were subjected to unpaired Student's t-test statistical analysis.

### Analysis of the neurochemical phenotype of PSA-NCAM immunoreactive puncta surrounding pyramidal cell somata

Sections processed for fluorescent immunohistochemistry were observed under a confocal microscope (Leica TCS-SPE) using a 63× oil objective. From each animal, 50 CaMKII-α expressing neurons from mPFC layers III and V displaying triangular-shaped soma were randomly selected from all the sections containing this cortical region (8–10 sections between Bregma +3,20 mm to −1,40 mm [17]). These cells were first identified using conventional fluorescence microscopy and then, Z-series of optical sections (0.5 µm apart) covering all its three-dimensional extension were acquired using sequential scanning mode. Stacks were processed with Zeiss LSM 5 Image Browser software at 4×-Zoom magnification. The soma profile of these neurons was drawn and puncta placed within an area 0.5 µm distal from the edge of this profile were analyzed. Puncta were defined as having an area not smaller than 0.15 and not larger than 2.5 µm^2^ [18]. The co-localization of PSA-NCAM and each of the different markers (GAD65/67, VGAT, VGLUT1, SYN, PV, CB and CR) was analyzed on five consecutive confocal planes from each selected neuron, in which the penetration of both antibodies was optimal. The percentage of puncta co-expressing PSA-NCAM and each marker was obtained for each neuron and mean ± S.E.M was determined. Puncta density values were also determined for each marker and expressed as number of puncta per micron of soma perimeter.

### Quantification of perisomatic puncta on mPFC pyramidal neurons

Sections processed for (1) CaMKII-α - PSA-NCAM, (2) CaMKII-α- GAD65/67 or (3) CaMKII-α - PV - SYN immunohistochemistries were observed and analyzed as described above, with some minor differences. In this case, six pyramidal neurons per animal from mPFC layers III and V were randomly selected from all sections containing this region and from each immunostaining. A total of 144 neurons were analyzed for each immunostaining.

Values of puncta density for PSA-NCAM, GAD65/67, PV and SYN were obtained from each neuron and expressed as number of puncta per micron of soma perimeter. For each experimental group, mean ± S.E.M. was determined and the resulting values were analyzed by unpaired Student's t-test (for the density of PSA-NCAM expressing puncta) or by one-way ANOVA, with the number of neurons as the “n”. Significant differences were further analyzed by Student-Newman-Keuls post-hoc test. The percentage of PV puncta co-expressing SYN was also obtained from each neuron and analyzed following the same procedure.

### Summary of statistical methods

For body weight analysis, body weight differences across the different time points (day 0, 7, 10 and 14) were subjected to one-way ANOVA tests followed, when appropriate, by multiple pair-wise comparisons with Bonferroni's correction (see **material and methods S1** and **table S1**).

In order to quantify PSA-NCAM, GAD67, SYN and NCAM neuropil immunoreactivities, optical densities were analyzed by one-way repeated measures ANOVA tests, where “region” (infralimbic, prelimbic, dorsal cingulate and ventral cingulate cortices) and “layer” (I, II, III, V, VI) were considered as within-subjects variables (repeated measures variables) and treatment (Endo-N/PPHT, Endo-N/Control Control/PPHT, Control/Control), as between-subjects variable (see materials and methods S1).

To analyze the differences on the total number of neurons expressing PSA-NCAM, CB, CR or PV between Control/Control and Control/PPHT groups, data were subjected to unpaired Student's t-test analysis.

In order to study whether PPHT treatment-induced differences on the neurochemical phenotype of PSA-NCAM expressing interneurons, the percentages of co-localization of PSA-NCAM with CB, CR or PV were determined for each animal from Control/Control and Control/PPHT group. Data were then subjected to unpaired Student's t-test analysis.

To analyze the differences on perisomatic innervation of mPFC pyramidal neurons after treatment, values of puncta density for PSA-NCAM, GAD65/67, PV and SYN and the percentages of PV puncta co-expressing SYN were obtained from each neuron (6 neurons from each of the 6 animals of the 4 experimental groups) and then analyzed by unpaired Student's t-test (for the density of PSA-NCAM expressing puncta) or by one-way ANOVA, with the number of neurons as the “n”. Significant differences were further analyzed by Student-Newman-Keuls post-hoc test.

## Results

### Body weight loss after PPHT treatment

From the day of surgery (day 0) to the onset of pharmacological treatment (day 7), no statistically significant differences were found on body weight between groups (p = 0.087). After PPHT treatment (from 7^th^ to 14^th^ day), animals showed a significant weight loss when compared with non-PPHT treated ones (Endo-N/PPHT vs. Endo-N/Control: p<0.0001; Control/PPHT vs. Control/Control: p = 0.0246). These decreases on body weight took place specifically from 7^th^ to 10^th^ day (Endo-N/PPHT vs. Endo-N/Control: p<0.0001; Control/PPHT vs. Control/Control: p = 0.005); from day 10^th^ to 14^th^, all groups increased equally their body weight (p = 0.322). Please, see **table S1** for detailed information.

### PSA-NCAM expressing elements in the mPFC: Pyramidal neuron somata in the mPFC are surrounded by puncta co-expressing inhibitory or synaptic markers

PSA-NCAM expression was found in three types of structures of the mPFC: (1) neuronal somata, (2) puncta in the neuropil, and (3) puncta surrounding the somata of pyramidal neurons.

Since PPHT modulates the expression of PSA-NCAM and molecules related to inhibitory neurotransmission in the mPFC and since many inhibitory contacts are established on the perisomatic region of pyramidal cortical neurons, we analyzed the presence and the phenotype of PSA-NCAM expressing puncta in this region.

**Figure 1:**
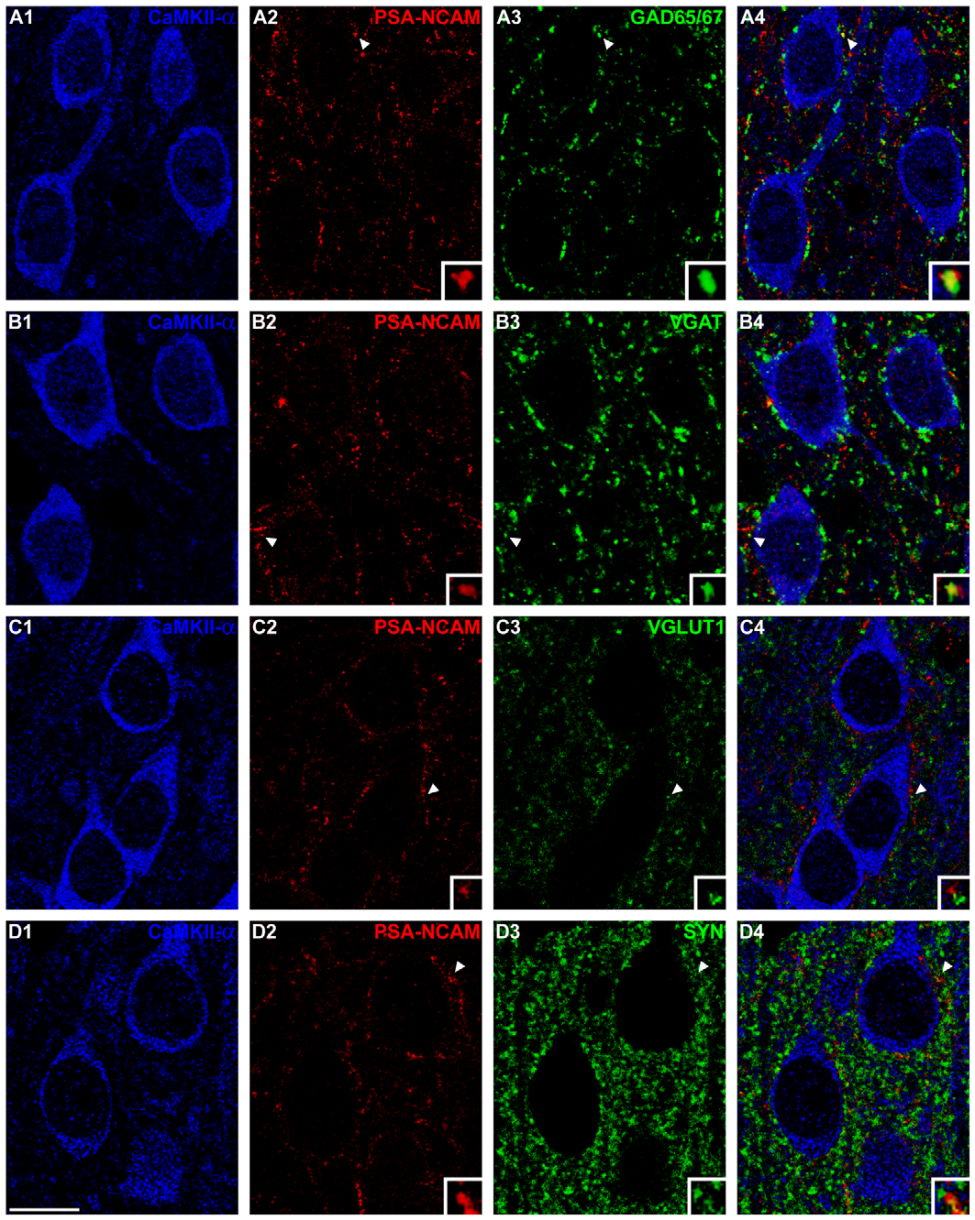
Confocal microscopic analysis of the neurochemical phenotype of PSA-NCAM immunoreactive puncta surrounding pyramidal cell somata in mPFC. (A) PSA-NCAM-expressing puncta co-localizing with GAD67 in the perisomatic region of CaMKII-α expressing neurons. (B) PSA-NCAM/VGAT double-labeled puncta surrounding CaMKII-α immunoreactive neurons. (C) Lack of co-localization between perisomatic PSA-NCAM-expressing puncta and VGLUT1-expressing puncta. (D) Perisomatic puncta co-expressing PSA-NCAM and SYN. All the images in this figure are taken from single confocal planes. Scale bar: 10 µm. Insets in the images are 5× enlargements of puncta marked with arroheads.

PSA-NCAM expressing puncta were found surrounding the somata of mPFC pyramidal neurons with a mean density of 0.368±0.014 puncta/µm (**table 2** **,** **figs. 1** **&** **2**). These puncta mainly co-expressed markers of inhibitory elements or synapses, such as GAD65/67 (36.073±2.704%; **table 2** **,** **fig. 1 A**) or VGAT (38.363±1.005%; **table 2** **,** **fig. 1 B**) and rarely co-expressed VGLUT1 (2.545±0.461%; **table 2** **,** **fig. 1 C**), a marker of excitatory synapses. The synaptic vesicle protein SYN was also frequently found in PSA-NCAM expressing puncta (34.338±1.858%; **table 2** **,** **fig. 1 D**).

**Figure 2:**
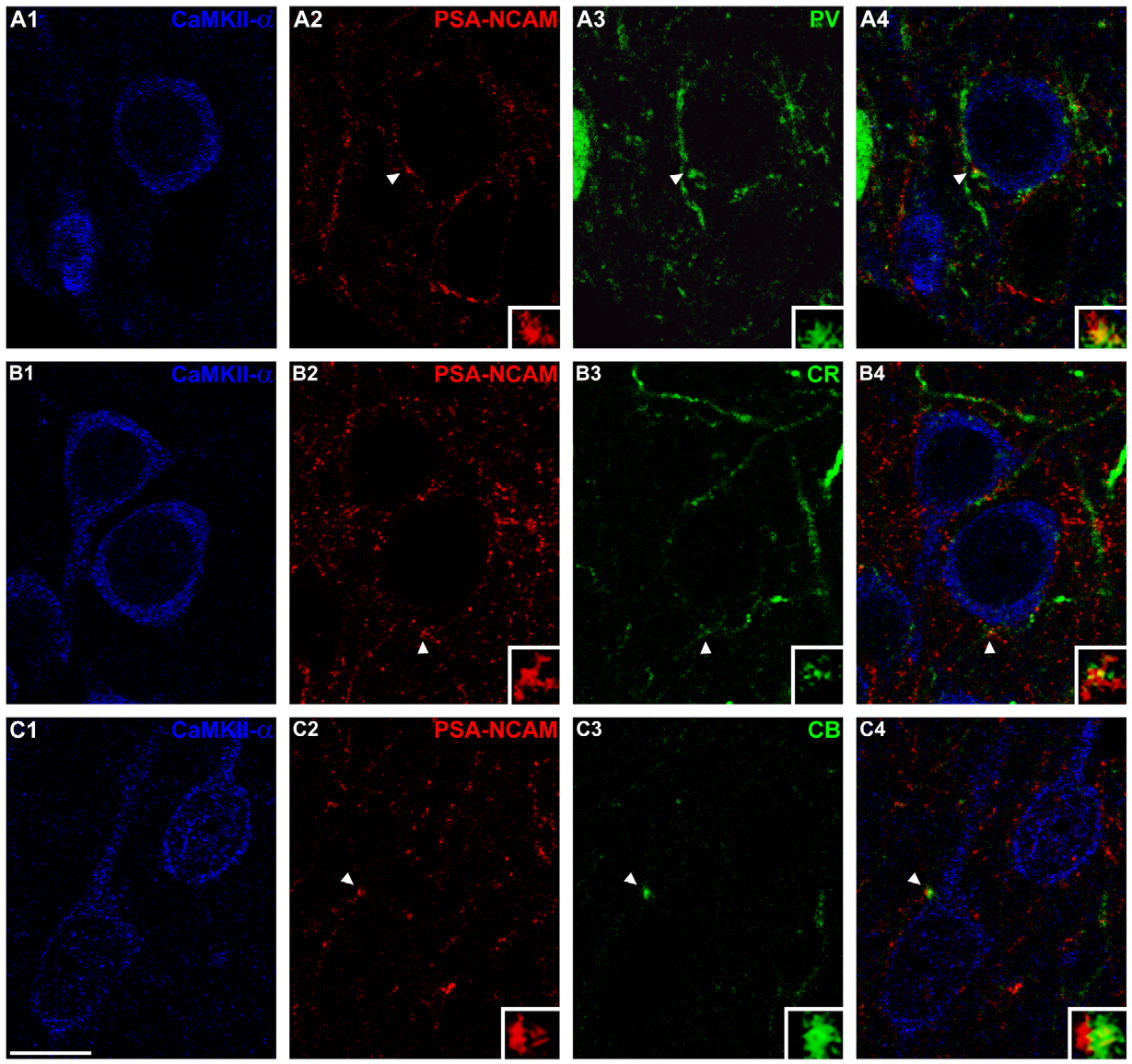
Confocal microscopic analysis of the co-expression of PV, CR and CB in PSA-NCAM immunoreactive puncta surrounding pyramidal cell somata in mPFC. (A) PSA-NCAM-expressing puncta co-expressing with PV in the perisomatic region of a neuron expressing CaMKII-α. (B) PSA-NCAM immunoreactive puncta co-expressing CR surrounding CaMKII-α expressing neurons. (C) Observe a CB-expressing puncta close to a PSA-NCAM expressing puncta in the perisomatic region of a pyramidal neuron. All the images in this figure are taken from single confocal planes. Scale bar: 10 µm. Insets in the images are 5× enlargements of puncta marked with arrowheads.

**Table 2:** Puncta surrounding pyramidal neurons somata.

Puncta immunoreactive for:	Puncta density (puncta/µm)(a)	Co-localization percentage (%)(b)
PSA-NCAM	0.368±0.014	
GAD65/67	0.330±0.040	
VGAT	0.565±0.003	
VGLUT1	0.171±0.006	
SYN	0.604±0.072	
CB	0.027±0.001	
CR	0.052±0.008	
PV	0.402±0.021	
PV– SYN	0.319±0.017	
PSA-NCAM – GAD65/67	0.093±0.018	36.073±2.704
PSA-NCAM – VGAT	0.184±0.006	38.363±1.005
PSA-NCAM – VGLUT1	0.004±0.001	2.545±0.461
PSA-NCAM – SYN	0.163±0.013	34.328±1.858
PSA-NCAM – CB	0.010±0.001	2.415±0.039
PSA-NCAM – CR	0.010±0.001	2.890±0.087
PSA-NCAM –PV	0.179±0.004	40.621±1.469
GAD65/67 – PSA-NCAM	0.093±0.018	28.114±2.891
VGAT – PSA-NCAM	0.184±0.006	32.948±0.869
VGLUT1 – PSA-NCAM	0.004±0.001	1.923±0.423
SYN –PSA-NCAM	0.163±0.013	26.831±0.908
CB – PSA-NCAM	0.010±0.001	42.977±3.690
CR – PSA-NCAM	0.010±0.001	20.624±1.122
PV – PSA-NCAM	0.179±0.004	43.331±3.622

(a) Puncta density data are expressed as mean ± s.e.m. of puncta expressing the named marker/s per micron of soma perimeter.

(b) Co-localization percentage of puncta expressing the first named marker which also expresses the second or second and third named marker/s (i.e. PSA-NCAM – GAD65/67 co-localization percentage: percentage of PSA-NCAM expressing puncta which also express GAD65/67). Data are expressed as mean± s.e.m.

Because PSA-NCAM expressing perisomatic puncta were shown to be mainly inhibitory, the co-expression in these puncta of the interneuron subpopulation markers PV, CR and CB was also studied. This analysis revealed that the highest percentage of co-localization occurred with PV (40.621±1.469%; **table 2** **,** **fig. 2 A**), whereas the co-localization with CR (2.890±0.087%; **table 2** **,** **fig. 2 B**) or CB (2.415±0.039%; **table 2** **,** **fig. 2 C**) was very low. The density of PV expressing perisomatic puncta was also substantially higher than that of puncta expressing CB or CR (see **table 2**).

All the results shown in the following sections have been summarized in **table 3**.

**Table 3:** Summary of results: differences from Control/Control group.

	CONTROL	ENDO-N	ENDO-N
	PPHT	CONTROL	PPHT
**Neuropil protein expression**			
PSA-NCAM	↑ ***		
SYN	↑ **	=	=
GAD67	↑ ***	=	=
NCAM	=	=	=
**PSA-NCAM expressing neurons**			
Number of neurons	↑ ***		
% of co-localization with CB	=		
% of co-localization with CR	=		
% of co-localization with PV	=		
**Number of neurons expressing CBPs**			
CB	↑ ***		
CR	↑ **		
PV	↑ **		
**Perisomatic puncta on pyramidal neurons**			
δ PSA-NCAM	↑ **		
δ GAD65/67	↑ ***	↑ ***	↑ ***
δ PV	↑ ***	↑ ***	↑ ***
δ SYN	=	↑ ***	↑ ***
% of PV puncta co-expressing SYN	↓ ***	↑ ***	↑ ***

No statistically significant differences ( = ) or statistically significant increases (↑) or decreases (↓) on the parameters measured, when compared with control/control group following the appropriate statistical analysis (see materials and methods section); p<0.05 (*), p<0.01 (**), p<0.001 (***).

Abbreviations: CBPs, Calcium-Binding Proteins; CB, calbindin-D28k; CR , calretinin; δ, density (number of puncta or spines/µm of soma perimeter); GAD67, 67 kDa isoform of the glutamate decarboxilase enzime, GAD65/67, both 65 and 67 kDa isoforms of the glutamate decarboxilase enzyme; NCAM, neural cell adhesion molecule; PSA-NCAM, polysialylated form of the NCAM; PV, parvalbumin; SYN, synaptophysin.

### PPHT treatment increases the number of interneurons expressing PSA-NCAM, CB, CR and PV in the mPFC, without affecting the neurochemical phenotype of PSA-NCAM expressing interneurons

**Figure 3:**
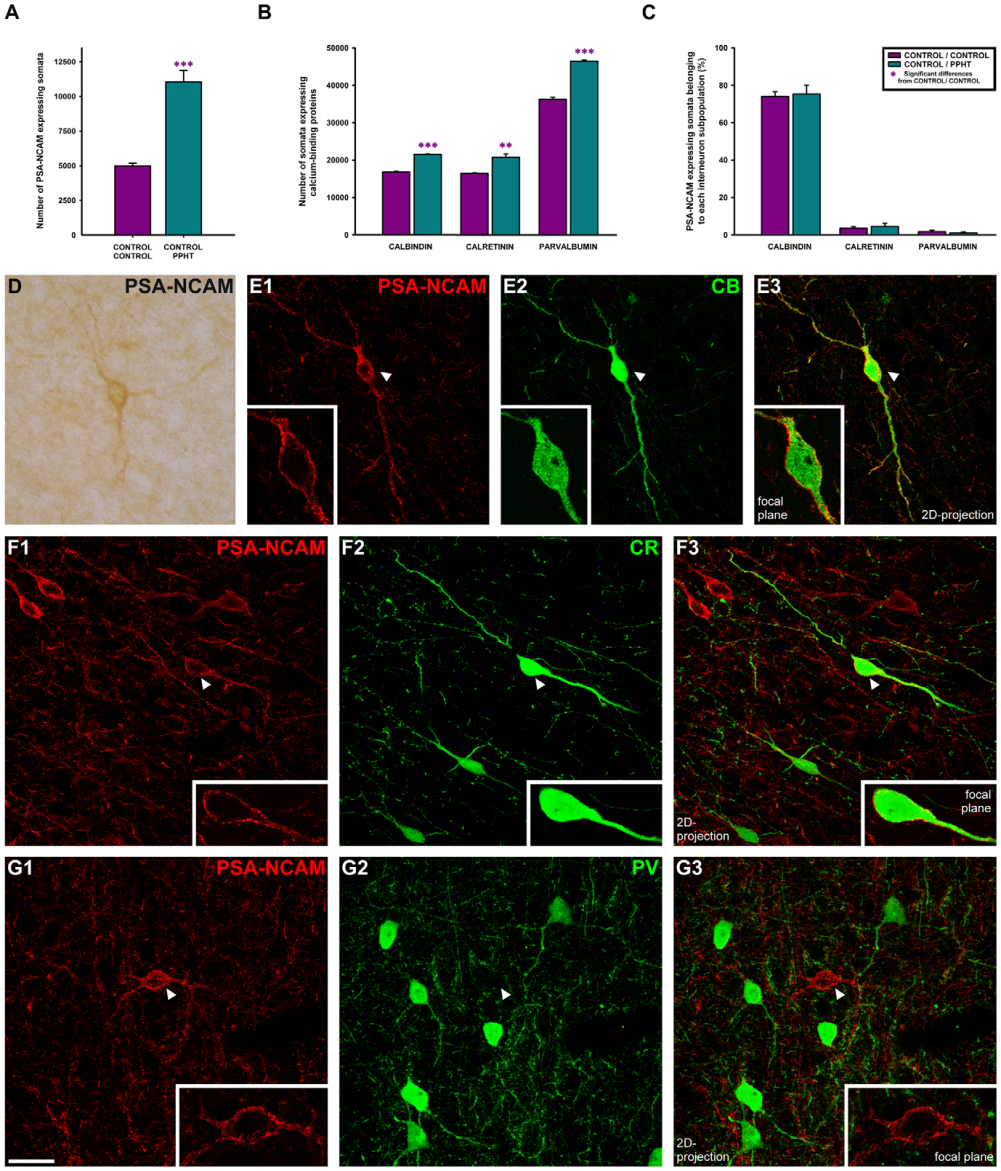
Quantification and confocal microscopic analysis of the neurochemical phenotype of PSA-NCAM expressing interneurons in mPFC after PPHT treatment. (A) Graph showing statistically significant differences in the number of PSA-NCAM expressing somata from control group after unpaired Student's t-test; p<0.001 (***). (B) Graph representing the changes in the number of somata belonging to different interneuron subpopulations (defined by the expression of the calcium-binding proteins CB, CR and PV) after PPHT treatment [p<0.01 (**); p<0.001 (***) after unpaired Student's expression] (C) Graph showing the percentages of PSA-NCAM expressing interneuron somata belonging to different interneuron subpopulations. No statistically significant differences from control group were observed after unpaired Student's t-tests (p>0.05 in all comparisons). (D) Multipolar neuron expressing PSA-NCAM in dorsal Cingulate Cortex (Cg1) layer V observed under conventional light microscopy. (E) PSA-NCAM interneuron in Cg1 layer V co-expressing CB. (F) PSA-NCAM/CR double labeled interneuron in ventral Cingulate Cortex (Cg2) layer V. (G) Lack of co-localization between PSA-NCAM (G1) and PV (G2) expressing neurons in Cg1 layer III. E–G images are 2D projections of focal planes located 1 µm apart. Scale bar: 10 µm. Insets in the images are 2× enlarged views taken from single confocal planes of the areas marked with arrowheads.

The number of PSA-NCAM expressing interneuron somata in the mPFC was higher in PPHT-treated animals than in non-treated ones (p = 0.0004) (**figs. 3A & D**). In particular, statistically significant differences were observed in layers III, V and VI of infralimbic, prelimbic, dorsal cingulate and ventral cingulate cortices (**fig. S1**).

The number of interneuron somata belonging to different interneuron subpopulations (defined by the expression of the calcium-binding proteins CB, CR and PV) was also increased after PPHT treatment in the mPFC (p<0.0001 for CB, p = 0.0044 for CR and p<0.0001 for PV) (figs. 3B). The same effect can also be observed in every region within the mPFC (see fig. S2 for further information about means and p-values).

Nevertheless, the percentages of PSA-NCAM expressing interneuron somata belonging to these interneuron subpopulations did not change after PPHT treatment (p>0.05 in all comparisons; **fig. 3 C**). Most PSA-NCAM interneurons co-expressed CB (74.0±2.6% in control group, 75.3±4.7% in PPHT treated group; **figs. 3 C & E**) and much lower percentages co-expressed CR (3.7±0.8% in control group, 4.5±1.7% in PPHT treated group; **figs. 3 C & F**) or PV (1.7±0.8% in control group, 1.0±0.6% in PPHT treated group; **figs. 3 C & G**).

### Endo-N injection blocks PPHT-induced increases in SYN and GAD67 expression in the mPFC neuropil, while NCAM expression is not affected by any of the treatments

For the neuropil expression of PSA-NCAM, SYN and GAD67, repeated-measures ANOVA test showed significant main effects of **treatment** (PSA-NCAM: F_3,14_ = 194.843, p<0.001; SYN: F_3,12_ = 8.665, p = 0.003; GAD67: F_3,11_ = 36.306, p<0.001; NCAM: F_3,15_ = 1.007, p = 0.001), **region** (PSA-NCAM: F_1.935,27.089_ = 13.89, p<0.001; SYN: F_3,36_ = 3.821, p = 0.018; GAD67: F_1.644,18.086_ = 12.014, p = 0.001) and **layer** (PSA-NCAM: F_1.120,15.685_ = 63.136, p<0.001; SYN: F_4,9_ = 176.617, p<0.001; GAD67: F_1.367,15.061_ = 178.867, p<0.001), so multiple pair wise-comparisons with Bonferroni's correction were performed for each immunostaining in order to compare treatment effect on the mPFC neuropil expression between pairs of experimental groups. However, for the neuropil expression of NCAM, repeated-measures ANOVA test showed significant main effects of region (F_1.855,31.539_ = 12.412, p<0.0001) and layer (F_1.979,33.635_ = 255.993, p<0.0001) but not treatment (F_3,15_ = 1.007, p = 0.417).

**Figure 4:**
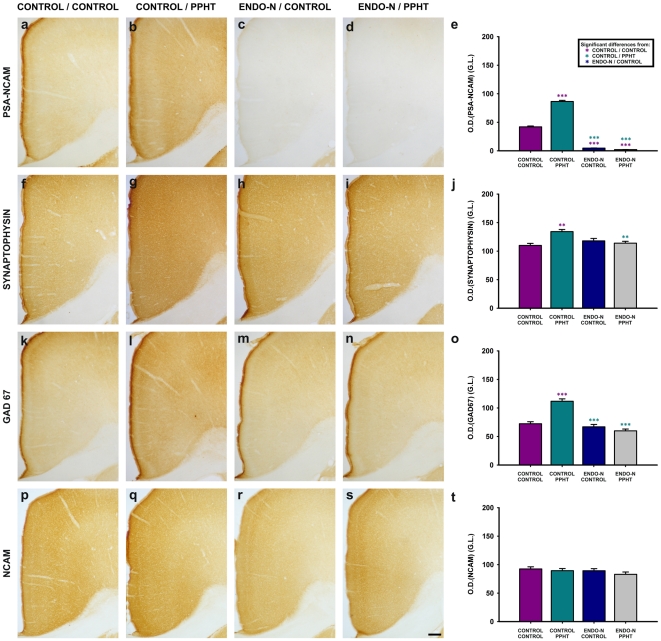
Panoramic views of the rat mPFC cortex and graphs showing the expression of PSA-NCAM (a–e), SYN (f–j), GAD67 (k–o) and NCAM (p–t) in the mPFC neuropil after Endo-N or PPHT treatments and their combination. Three sections per animal were examined under bright-field illumination, homogeneously lighted and digitalized using a CCD camera at 20× magnification. Grey levels were converted to optical densities (OD) using Image J software (NIH). Detailed description of this method of quantification can be found in **material and methods S1**. Note in panel c & d, the lack of PSA-NCAM expression in the cingulate cortex, but not in the striatum, demonstrating the effectiveness of Endo-N treatment. Asterisks in bars indicate statistically significant differences between groups (see graph legend) after univariate repeated measures ANOVA followed by multiple pair-wise comparisons with Bonferroni's correction; p<0.05 (*), p<0.01 (**), p<0.001 (***). Scale bar: 100 µm.

Regarding treatment effect and, in consonance with our previous results [12], 7 days of PPHT treatment induced statistically significant increases in PSA-NCAM (p<0.001; **fig. 4 a, b & e**), SYN (p = 0.003; **fig. 4 f, g & j**) and GAD67 (p<0.001; **fig. 4 k, l & o**) expression in the mPFC neuropil. These effects of PPHT on SYN and GAD67 expression were prevented when PSA was removed one week before the onset of the PPHT treatment [Control/Control vs. Endo-N/PPHT: p = 1.000 (SYN), p = 0.139 (GAD67); Endo-N/Control vs. Endo-N/PPHT: p = 1.000 (SYN and GAD67)] **(** **fig. 4 h-j & m–o)**. PSA-NCAM expression in the mPFC remained undetectable 14 days after Endo-N injection (**fig. 4 c–e**).

Endo-N administration by itself did not induce changes in GAD67 or SYN expression in the mPFC neuropil [Endo-N/Control vs. Control/Control: p = 1.000 for GAD67 and SYN] (**fig. 4 j & o**).

NCAM expression in the mPFC neuropil was not affected by any of the treatments [Control/Control vs. Control/PPHT: p = 1.000; Control/Control vs. Endo-N/Control: p = 1.000; Control/Control vs. Endo-N/PPHT: p = 0.643; Control/PPHT vs. Endo-N/PPHT: p = 1.000] (**fig. 4 p–t**).

Three-way interaction (**region×layer×treatment**) on the repeated measures ANOVA test was also significant (p<0.05) for the neuropil expression of PSA-NCAM, SYN and GAD67 but not for NCAM (PSA-NCAM: F_11.716,54.675_ = 2.626, p = 0.008; SYN: F_12,3_ = 11.002, p = 0.036; GAD67: F_14.621,53.610_ = 2.021, p = 0.032; NCAM: F_13.680,77.519_ = 0.794, p = 0.670), so multiple pair-wise comparison with Bonferroni's correction were performed only for PSA-NCAM, SYN and GAD67, in order to analyze in which specific layer from each region inside the mPFC there were differences between groups. The results indicate that the same effects described above for PSA-NCAM, SYN and GAD67 expression, considering the whole mPFC neuropil, can also be observed in most layers of every region within the mPFC (infralimbic, prelimbic, dorsal cingulate and ventral cingulate cortices) (see **figs. S4, S5, S6**, for further information about means and p-values).

### Effects of PPHT and Endo-N treatments on perisomatic puncta of mPFC pyramidal neurons

#### PPHT treatment increases the density of PSA-NCAM expressing perisomatic puncta

**Figure 5:**
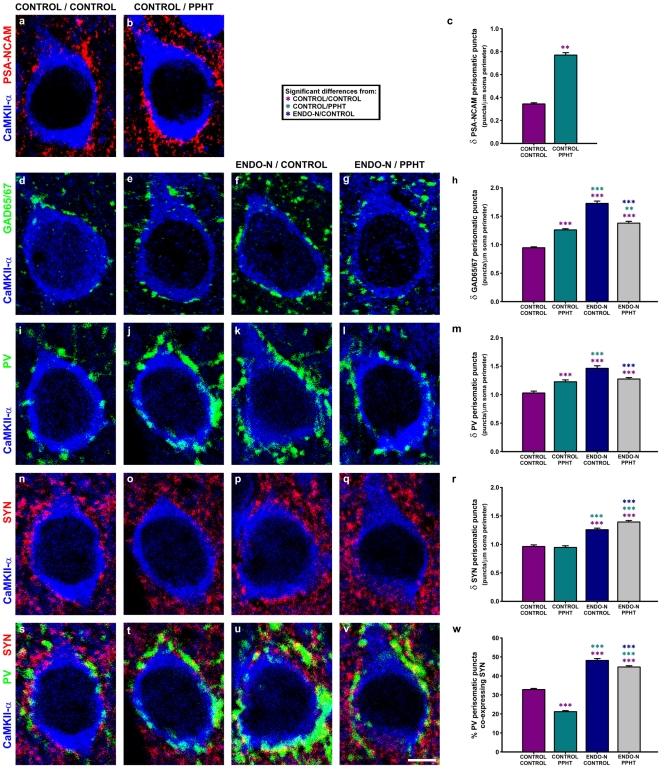
Confocal microscopic analysis of PSA-NCAM, GAD65/67, PV and SYN expressing puncta in the perisomatic region of mPFC pyramidal neurons after Endo-N or PPHT treatments and their combination. Focal planes of pyramidal neuron somata (immunolabeled for CaMKII-α) showing the changes in the perisomatic density of PSA-NCAM (a–b), GAD65/67 (d–g), PV (i–l) and SYN (n–q) expressing puncta after the different treatments, as can be observed in graphs (c), (h), (m) and (r), respectively. (s–w) Focal planes and graph showing the changes on the percentage of PV expressing puncta co-expressing SYN after treatment. Asterisks in bars indicate statistically significant differences between groups (see graph legend) after unpaired Student's t-test (c) or one-way ANOVA (h, m, r, w) followed by Bonferroni's correction; p<0.05 (*), p<0.01 (**), p<0.001 (***).

PPHT treatment induces a statistically significant 123.8% increase in the density of PSA-NCAM expressing puncta surrounding pyramidal neuron somata (p = 0.0023; **fig. 5 a–c**), which is in line with the effects described above in PSA-NCAM expressing somata and neuropil puncta.

#### PPHT and/or Endo-N treatments increase the density of inhibitory perisomatic puncta

Both PPHT and Endo-N treatments when administered by themselves induced statistically significant increases (p<0.0001 in all cases) in the density of GAD65/67 (33.2% and 82.54%, respectively; **fig. 5 d–h**) and PV (19.2% and 38.59%, respectively **fig. 5 i–m**) expressing puncta in the perisomatic region of pyramidal neurons. However, the combination of both treatments induced statistically significant but lower increases than those observed after Endo-N treatment alone (45.8% increase, p<0.0001 for GAD65/67; 23.8% increase, p<0.0001 for PV; **figs. 5 g–h & l–m**).

#### Endo-N, by itself or administered before PPHT treatment, increases the density of SYN perisomatic puncta

Endo-N administration by itself induced a statistically significant 30.5% increase in the density of SYN expressing puncta surrounding pyramidal neuron somata (p<0.0001). Increases were even higher when PPHT was administered after Endo-N injection (44.7% increase; p<0.0001 compared with Control/Control; p = 0.0008 compared with Endo-N/Control). By contrast, PPHT treatment alone did not change the density of SYN perisomatic puncta (p = 0.6660) (**fig. 5 n–r**).

#### PPHT decreases and Endo-N increases the percentage of PV perisomatic puncta co-expressing SYN

After PPHT treatment, 21.20±0.53% of PV expressing puncta surrounding pyramidal neuron somata were found to co-express SYN, which implied a small but significant decrease of this percentage (p<0.0001) when compared with that of the control group (32.82±0.56%). Conversely, Endo-N administration induced a statistically significant increase of this percentage, reaching 48.16±0.99% (p<0.0001). When Endo-N was administered before PPHT treatment, 44.72±0.65% of PV puncta co-expressed SYN, a percentage significantly lower than that of Endo-N/Control group (p = 0.0006) but still higher than that of the Control/Control group (p<0.0001) (**fig. 5 s–w**).

## Discussion

### Effects of D2R agonist treatment on mPFC neuronal circuitry

Our results are in accordance with previous findings demonstrating that chronic PPHT treatment increases GAD67 and SYN expression [12] and synaptic density [19] in the mPFC. These effects are probably mediated by dopamine through D2R, because D2R antagonists and dopamine depletion produce opposite effects [12]. The PPHT-induced upregulation of GAD67 expression in the neuropil probably reflects an increase in the number of inhibitory synapses on pyramidal neurons, since the number of puncta expressing these GABA-synthesizing enzymes also increases in the and perisomatic region of these cells. A direct effect of PPHT on these inhibitory structures is likely, because D2R are expressed in interneurons [20] and activation of D2R attenuates excitatory synaptic transmission in the adult PFC, involving GABA release by local inhibitory cells [21]. It is possible that this activation occurs mainly on basket interneurons, because D2R are particularly abundant in these cells [22] and specific agonists of D2R activate them [23]. Supporting this hypothesis, we have also detect an increase in the density of puncta expressing PV, a calcium binding protein expressed by many fast spiking/basket interneurons, in the perisomatic region of pyramidal neurons.

Our results on the effects of PPHT may have implications in the understanding of the molecular bases of schizophrenia and major depression, since decreased levels of dopamine [24], [25] and D2R [26], [27], as well as deficits in GABAergic neurotransmission and PV expressing interneurons have been found in the PFC of patients and animal models of these disorders [28], [29].

### Effects of PPHT on PSA-NCAM expression

PSA-NCAM expression in the mPFC is exclusively located in somata and neuropil elements belonging to inhibitory neurons, both in rodents and in humans [9], [11], [30]. Consequently, PPHT-induced increases in PSA-NCAM expression in the mPFC should affect primarily these inhibitory neurons rather than principal neurons, which do not express PSA-NCAM [11]. In fact, PSA-NCAM expressing interneurons in the mPFC express D2R and dopaminergic fibers are frequently found in their vicinity [12].

PSA-NCAM expressing neuronal somata in the mPFC of adult rats correspond to different interneuronal subpopulations (attending to their expression of different calcium binding proteins and neuropeptides): the majority of these cells express CB, few of them express CR and almost none of them expressed PV [9], [11]. A very similar neurochemical phenotype has been found in PSA-NCAM expressing neurons in the mPFC of adult humans [30], mice [11], [31] and cats [32], with only minor differences in the percentages across species. The number of PSA-NCAM expressing interneurons in this region is stable over lifetime in rats [10], but pharmacological manipulations of monoaminergic neurotransmission (present work and [10]) can decrease or increase it, indicating that inhibitory neurons in the mPFC can probably stop expressing, express “de novo” or re-express PSA-NCAM. Nevertheless, we have shown that the percentages of PSA-NCAM expressing interneurons belonging to different interneuron subpopulations did not change after D2R agonist treatment. However, the total number of interneuron somata expressing each of the three Calcium-binding proteins analyzed is increased after PPHT treatment. These results are in agreement with those describing a PPHT-induced increase of GAD67 expression and indicate that the activity of different populations of interneurons can be affected by PPHT.

PSA-NCAM expressing puncta that surround pyramidal neurons in the mPFC of adult rats also seem to correspond to inhibitory and synaptic elements, as it has been described before for neocortical neuropil elements expressing this molecule [11]. Surprisingly, despite the fact that it is extremely rare to find this molecule in neuronal somata expressing PV [9], [11], we have found that co-expression of PV and PSA-NCAM is common in perisomatic puncta on pyramidal neurons. This suggests that in PV expressing interneurons PSA-NCAM may have a restricted expression pattern, similar to that of mature hippocampal granule neurons, which only express it in their axons, but not in their somata or dendrites [33]. Moreover, its expression in PV expressing puncta may be regulated by the manipulation of dopamine neurotransmission in the mPFC, as indicated by the present data.

Given its anti-adhesive properties, PSA-NCAM expression in neurons has been usually linked to plastic events, such as dendritic or spine remodeling and synaptogenesis [6]–[8], [34]. However, a non-excluding insulating role for PSA-NCAM, preventing, totally or partially, the establishment of synapses should not be discarded. In fact, taking into account its steric effects [35], the addition of PSA to NCAM in a synaptic contact should prevent or profoundly affect normal neurotransmission. Consequently, the expression of PSA-NCAM in certain neuropil elements and especially in perisomatic puncta on pyramidal cells may indicate that these structures are not establishing functional synaptic contacts. In connection with this idea, we have recently found that PSA-NCAM expressing interneurons have reduced synaptic input, reduced spine density and are less arborized than neighboring interneurons lacking PSA-NCAM, which suggests that they are partially disconnected from cortical circuitry [11]. The effects of PPHT on the expression of PSA-NCAM may have been produced by an increase of the polysialylation on preexisting NCAM (before its incorporation to the plasma membrane) or by the synthesis of new NCAM that became polysialylated. Since we have not detected changes in total NCAM expression, the first possibility is less likely. Endo-N treatment also failed to modify NCAM expression, suggesting that the removal of PSA does not affect the synthesis of NCAM, at least at the time points analyzed in our study.

### Effects of PSA depletion from the mPFC

Removal of PSA from the mPFC completely blocks the effects of PPHT treatment on SYN and GAD65/67 expression in the neuropil. Consequently, the appearance of new structures belonging to interneurons and new synapses (presumably inhibitory ones) may need the expression of PSA-NCAM. The anti-adhesive properties of this molecule may favor the extension/formation of neurites and synapses from certain interneurons in a similar way to what has been described in hippocampal granule neurons [36].

Although PSA depletion by itself does not affect the expression of SYN and GAD65/67 in the mPFC neuropil, it induces dramatic effects in the perisomatic region of pyramidal neurons, increasing the density of perisomatic puncta expressing GAD65/67, PV and SYN, as well as the percentage of PV puncta that co-express SYN. A possible explanation for these effects is that the removal of PSA from NCAM may activate some perisomatic synapses, which were previously blocked by the presence of PSA. Consequently, the expression of molecules related to active inhibitory neurotransmission, such as SYN, GAD65/67 and PV, is increased and more puncta become detectable. These results are in agreement with a previous study on the developing visual cortex, in which PSA depletion induced precocious maturation of perisomatic innervation by basket interneurons, resulting in enhanced inhibitory synaptic transmission [18]. Thus, the presence of PSA-NCAM in control adult mPFC may act as a regulator of perisomatic inhibitory innervation.

**Figure 6:**
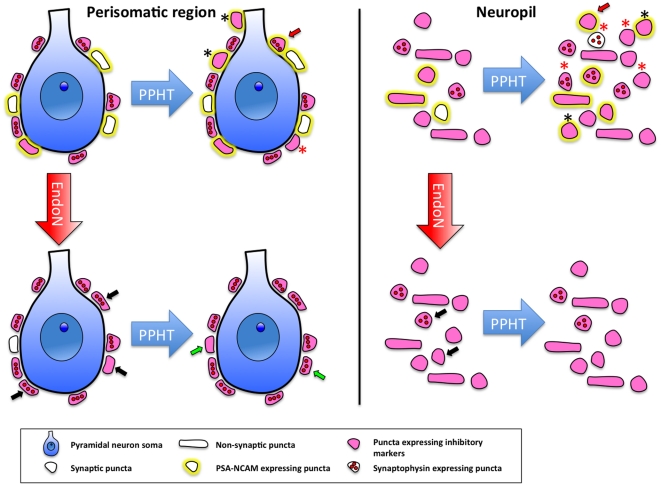
Schematic drawing of the hypothetic effects of PPHT and/or Endo-N administration in the perisomatic region of pyramidal neurons and in the neuropil of the medial prefrontal cortex. **Perisomatic region**: **PPHT** induces the appearance of new inhibitory contacts: some of them express PSA-NCAM (black asterisks), but some others may have already lost PSA-NCAM expression (red asterisks). Expression of PSA-NCAM may also be induced in preexisting inhibitory contacts (red arrow). It is possible that some of these PSA-NCAM expressing puncta were not functional synapses and this may be why we do not detect changes in the number of synaptophysin expressing puncta and there are decreases in the percentage of parvalbumin positive puncta that co-express synaptophysin. **Endo-N** depletes PSA-NCAM from the perisomatic region and promotes the maturation of contacts that previously expressed PSA-NCAM, inducing the expression of inhibitory markers (parvalbumin/GAD) and/or synaptophysin (black arrows), leading also to an increase of the percentage of parvalbumin puncta co-expressing synaptophysin. **When PPHT is administered after Endo-N**, most puncta remain stable, although some of them may continue with the maturation process initiated by PSA depletion and/or promoted by PPHT (green arrows). **Neuropil**: Note that the proportion of PSA-NCAM expressing puncta is more reduced than in the perisomatic region of pyramidal neurons and that many of these puncta do not correspond to synaptic boutons. **PPHT** induces the appearance of inhibitory puncta; some of them express PSA-NCAM (black asterisks) and some of them may have already lost its expression (red asterisks). Some of these newly generated structures may have matured faster than in the perisomatic region and have started to express synaptophysin. **Endo-N** administration may have little impact on neuropil because of the low proportion of PSA-NCAM expressing puncta in this region and because the nature of these puncta is different: most perisomatic puncta should correspond to presynaptic boutons contacting the pyramidal cell body, while in the neuropil many of these structures correspond to dendritic and axonal processes and not to synaptic contacts. The expression of inhibitory markers (parvalbumin/GAD) and/or synaptophysin would only be induced in some scarce puncta (black arrows). **The administration of PPHT after Endo-N** does not produce any effect, because the presence of PSA-NCAM may be absolutely necessary to induce the changes mediated by PPHT in this region.

A puzzling aspect of our study is why the effects of PSA removal on puncta expressing markers of general and inhibitory synapses are important in the perisomatic region of pyramidal neurons, but virtually absent in the neuropil of the mPFC. To understand these differences, first it has to be considered that the density of puncta expressing PSA is higher in the former region than in the latter. Consequently, changes induced by PSA depletion will have a higher impact and will be easily detected in the perisomatic region. It is also important to consider that the nature of puncta is different in these two regions. While most perisomatic puncta should correspond to presynaptic boutons contacting the pyramidal cell body, in the neuropil many of these structures correspond to dendritic and axonal processes and not mainly to synaptic contacts. Consequently, the impact of PSA depletion on proteins associated to synapses should be more reduced. A scheme depicting the hypothetic effects of the treatments used in our study on the perisomatic region of pyramidal neurons and in the neuropil of the mPFC is shown in **figure 6**.

The changes induced by PSA depletion or by alterations in the degree of NCAM polysialylation, may not only be caused by the regulation of NCAM adhesive properties, but also by the influence of PSA on NCAM-mediated signaling (see [7] for review). Moreover, interference on NCAM may also impair dopaminergic neurotransmission, since NCAM regulates the trafficking, internalization and degradation of D2R [37]. Interestingly, there is evidence for a relationship between dysregulation of NCAM and its posttranslational modifications and the neural abnormalities found in different mood disorders [38]. Moreover, one of the enzymes responsible for NCAM polysialylation is a candidate susceptibility gene for schizophrenia and bipolar disorder [39], [40] and PSA-NCAM expression is altered in the brain of schizophrenics [41]. Our results showing the involvement of PSA-NCAM in the plasticity induced by D2R manipulation also support the idea that altered PSA-NCAM expression may participate in the etiology of these psychiatric disorders.

## Supporting Information

Figure S1 **Graphs showing the changes in the number of PSA-NCAM immunoreactive neurons after PPHT treatment.** (A) Infralimbic cortex; (B) Prelimbic cortex; (C) Dorsal cingulate cortex; (D) Ventral cingulate cortex. Asterisks in bars indicate statistically significant differences from control group after repeated measures ANOVA followed by multiple pair-wise comparisons with Bonferroni's correction; p<0.05 (*), p<0.01 (**), p<0.001 (***). Roman numbers indicate cortical layers.
(TIF)

Figure S2 **Graphs showing the changes in the number of neurons expressing CB, CR or PV after PPHT treatment.** (A) Infralimbic cortex; (B) Prelimbic cortex; (C) Dorsal cingulate cortex; (D) Ventral cingulate cortex. Asterisks in bars indicate statistically significant differences from control group after repeated measures ANOVA followed by multiple pair-wise comparisons with Bonferroni's correction; p<0.05 (*), p<0.01 (**),p<0.001 (***).
(TIF)

Figure S3 **Panoramic views of the rat mPFC cortex showing the distribution of PSA-NCAM (B, F), SYN (C, G) and GAD67 (D, H) immunoreactivity in the neuropil.** Pictures A–D show the infralimbic (IL) and prelimbic (PrL) regions of the rat mPFC and pictures E–H, the dorsal (Cg1) and ventral cingulate cortices (Cg2). (A, E) Nissl staining was used for determining layer boundaries within mPFC regions, based on cytoarchitectural differences across these layers. Roman numbers indicate cortical layers. Scale bar: 200 µm.
(TIF)

Figure S4 **Graphs representing the changes in PSA-NCAM neuropil expression after Endo-N and/or PPHT treatments.** (A) Infralimbic cortex; (B) Prelimbic cortex; (C) Dorsal cingulate cortex; (D) Ventral cingulate cortex. Asterisks in bars indicate statistically significant differences between groups (see graph legend) after univariate repeated measures ANOVA followed by multiple pair-wise comparisons with Bonferroni's correction; p<0.05 (*), p<0.01 (**), p<0.001 (***). Roman numbers indicate cortical layers.
(TIF)

Figure S5 **Graphs showing the changes in SYN neuropil expression after Endo-N and/or PPHT treatments.** (A) Infralimbic cortex; (B) Prelimbic cortex; (C) Dorsal cingulate cortex; (D) Ventral cingulate cortex. Asterisks in bars indicate statistically significant differences between groups (see graph legend) after univariate repeated measures ANOVA followed by multiple pair-wise comparisons with Bonferroni's correction; p<0.05 (*), p<0.01 (**), p<0.001 (***). Roman numbers indicate cortical layers.
(TIF)

Figure S6 **Graphs representing the changes in GAD67 neuropil expression after Endo-N and/or PPHT treatments.** (A) Infralimbic cortex; (B) Prelimbic cortex; (C) Dorsal cingulate cortex; (D) Ventral cingulate cortex. Asterisks in bars indicate statistically significant differences between groups (see graph legend) after univariate repeated measures ANOVA followed by multiple pair-wise comparisons with Bonferroni's correction; p<0.05 (*), p<0.01 (**), p<0.001 (***). Roman numbers indicate cortical layers.
(TIF)

Table S1 **Body weight analysis.** Body weight data of all experimental animals in the day of surgery (day 0), before the onset of pharmacological treatment (day 7), in the middle of pharmacological treatment (day 10) and at the end of the experiment (day 14). Body weight differences across the different time points (day 0, 7, 10 and 14) were calculated ant then analyzed by one-way ANOVA tests (Inter-groups) followed, when appropriate, by multiple pair-wise comparisons with Bonferroni's correction. No statistically significant differences [n.s (p>0.05)] or statistically significant differences [p<0.05(*), p<0.01 (**), p<0.001 (***)] between groups.
(DOC)

Materials and Methods S1 **Supporting materials and methods.**
(DOC)
