# Supplementary figures and images for: Polysialic Acid Is Required for Dopamine D2 Receptor-Mediated Plasticity Involving Inhibitory Circuits of the Rat Medial Prefrontal Cortex
Source: PLoS One. 2011 Dec 28;6(12):e29516. doi: 10.1371/journal.pone.0029516 (PMC3247286; doi:10.1371/journal.pone.0029516)

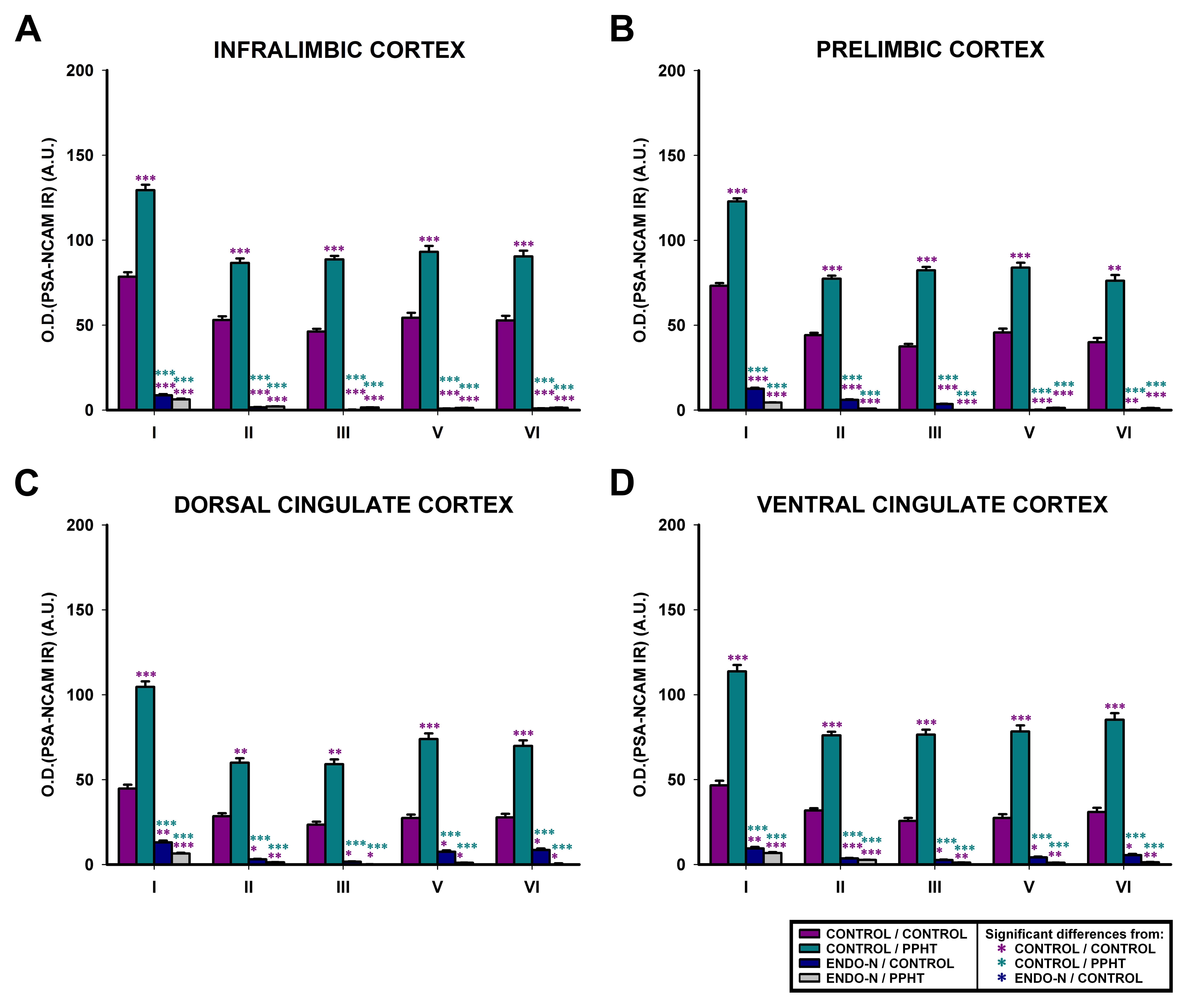

Supplement: Figure S1 — Graphs showing the changes in the number of PSA-NCAM immunoreactive neurons after PPHT treatment. (A) Infralimbic cortex; (B) Prelimbic cortex; (C) Dorsal cingulate cortex; (D) Ventral cingulate cortex. Asterisks in bars indicate statistically significant differences from control group after repeated measures ANOVA followed by multiple pair-wise comparisons with Bonferroni's correction; p<0.05 (*), p<0.01 (**), p<0.001 (***). Roman numbers indicate cortical layers. (TIF) [file pone.0029516.s001.tif]

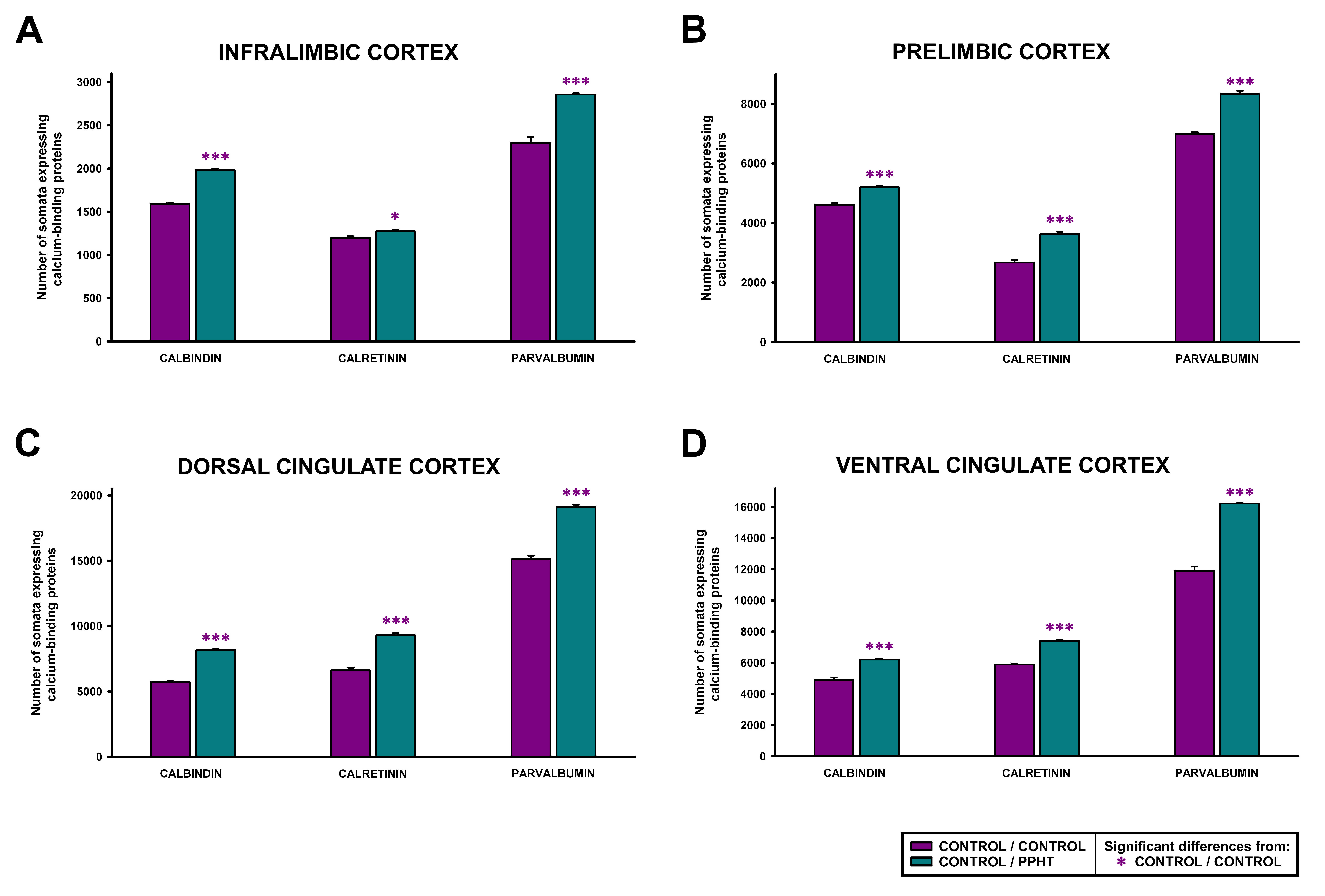

Supplement: Figure S2 — Graphs showing the changes in the number of neurons expressing CB, CR or PV after PPHT treatment. (A) Infralimbic cortex; (B) Prelimbic cortex; (C) Dorsal cingulate cortex; (D) Ventral cingulate cortex. Asterisks in bars indicate statistically significant differences from control group after repeated measures ANOVA followed by multiple pair-wise comparisons with Bonferroni's correction; p<0.05 (*), p<0.01 (**),p<0.001 (***). (TIF) [file pone.0029516.s002.tif]

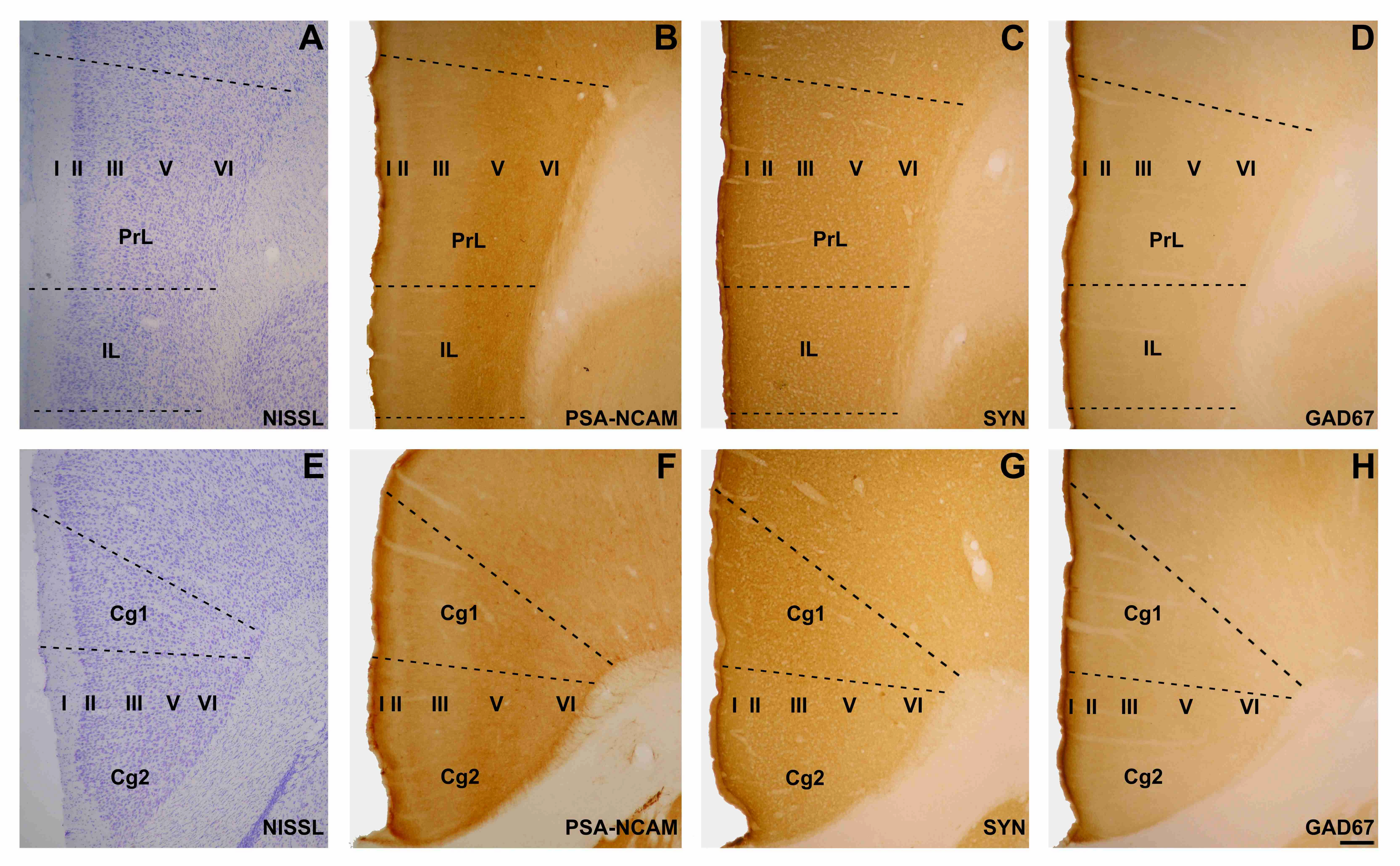

Supplement: Figure S3 — Panoramic views of the rat mPFC cortex showing the distribution of PSA-NCAM (B, F), SYN (C, G) and GAD67 (D, H) immunoreactivity in the neuropil. Pictures A–D show the infralimbic (IL) and prelimbic (PrL) regions of the rat mPFC and pictures E–H, the dorsal (Cg1) and ventral cingulate cortices (Cg2). (A, E) Nissl staining was used for determining layer boundaries within mPFC regions, based on cytoarchitectural differences across these layers. Roman numbers indicate cortical layers. Scale bar: 200 µm. (TIF) [file pone.0029516.s003.tif]

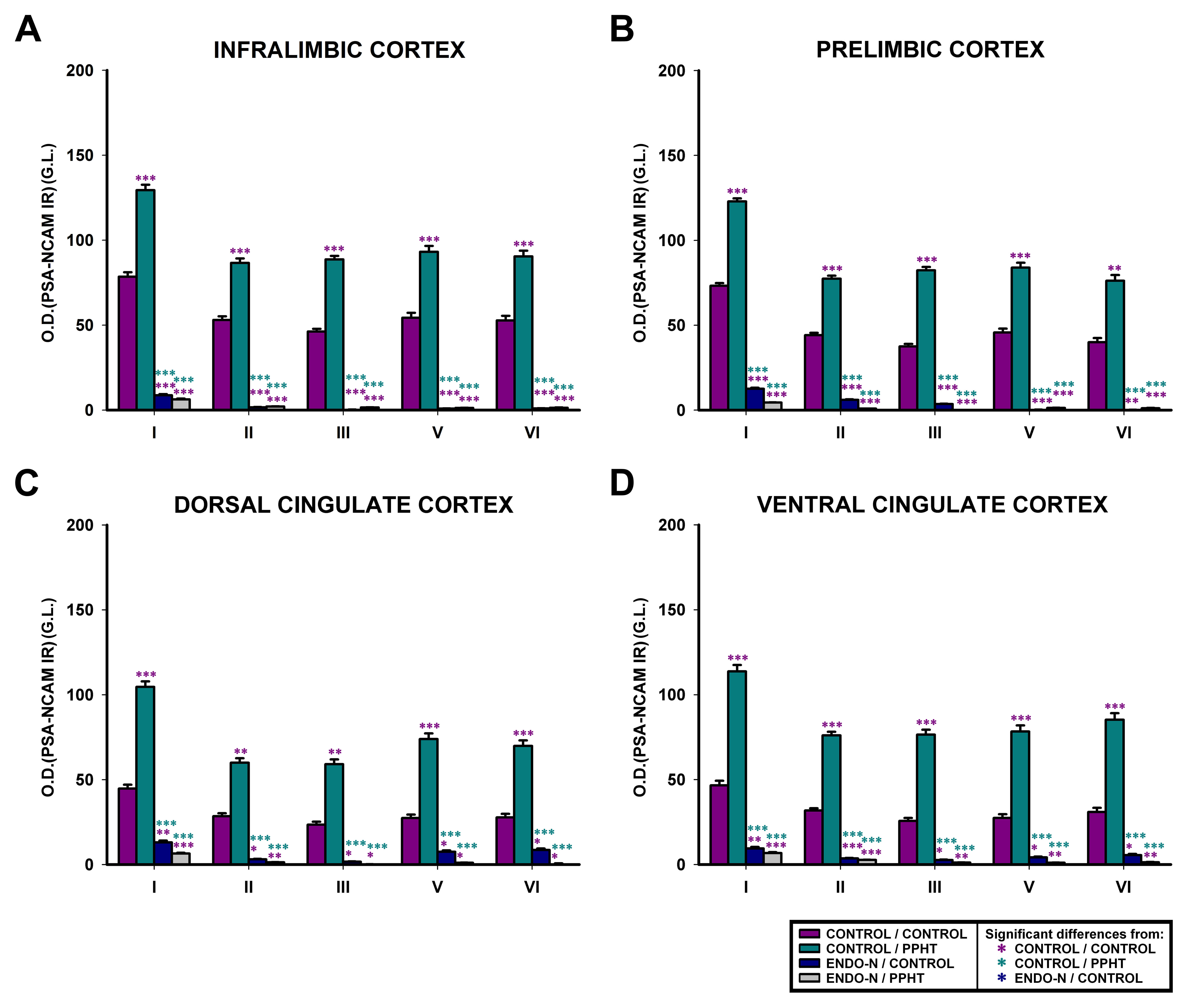

Supplement: Figure S4 — Graphs representing the changes in PSA-NCAM neuropil expression after Endo-N and/or PPHT treatments. (A) Infralimbic cortex; (B) Prelimbic cortex; (C) Dorsal cingulate cortex; (D) Ventral cingulate cortex. Asterisks in bars indicate statistically significant differences between groups (see graph legend) after univariate repeated measures ANOVA followed by multiple pair-wise comparisons with Bonferroni's correction; p<0.05 (*), p<0.01 (**), p<0.001 (***). Roman numbers indicate cortical layers. (TIF) [file pone.0029516.s004.tif]

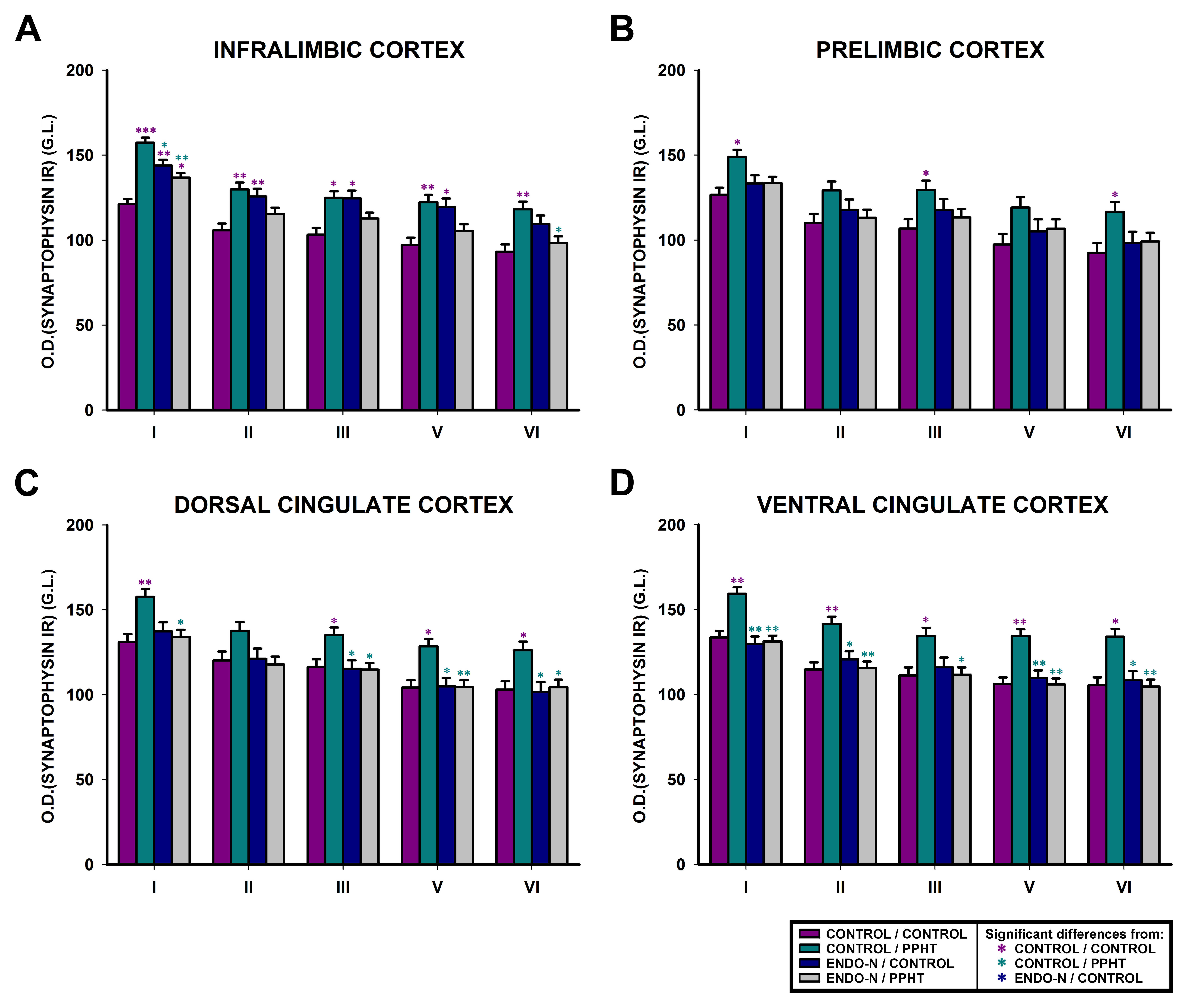

Supplement: Figure S5 — Graphs showing the changes in SYN neuropil expression after Endo-N and/or PPHT treatments. (A) Infralimbic cortex; (B) Prelimbic cortex; (C) Dorsal cingulate cortex; (D) Ventral cingulate cortex. Asterisks in bars indicate statistically significant differences between groups (see graph legend) after univariate repeated measures ANOVA followed by multiple pair-wise comparisons with Bonferroni's correction; p<0.05 (*), p<0.01 (**), p<0.001 (***). Roman numbers indicate cortical layers. (TIF) [file pone.0029516.s005.tif]

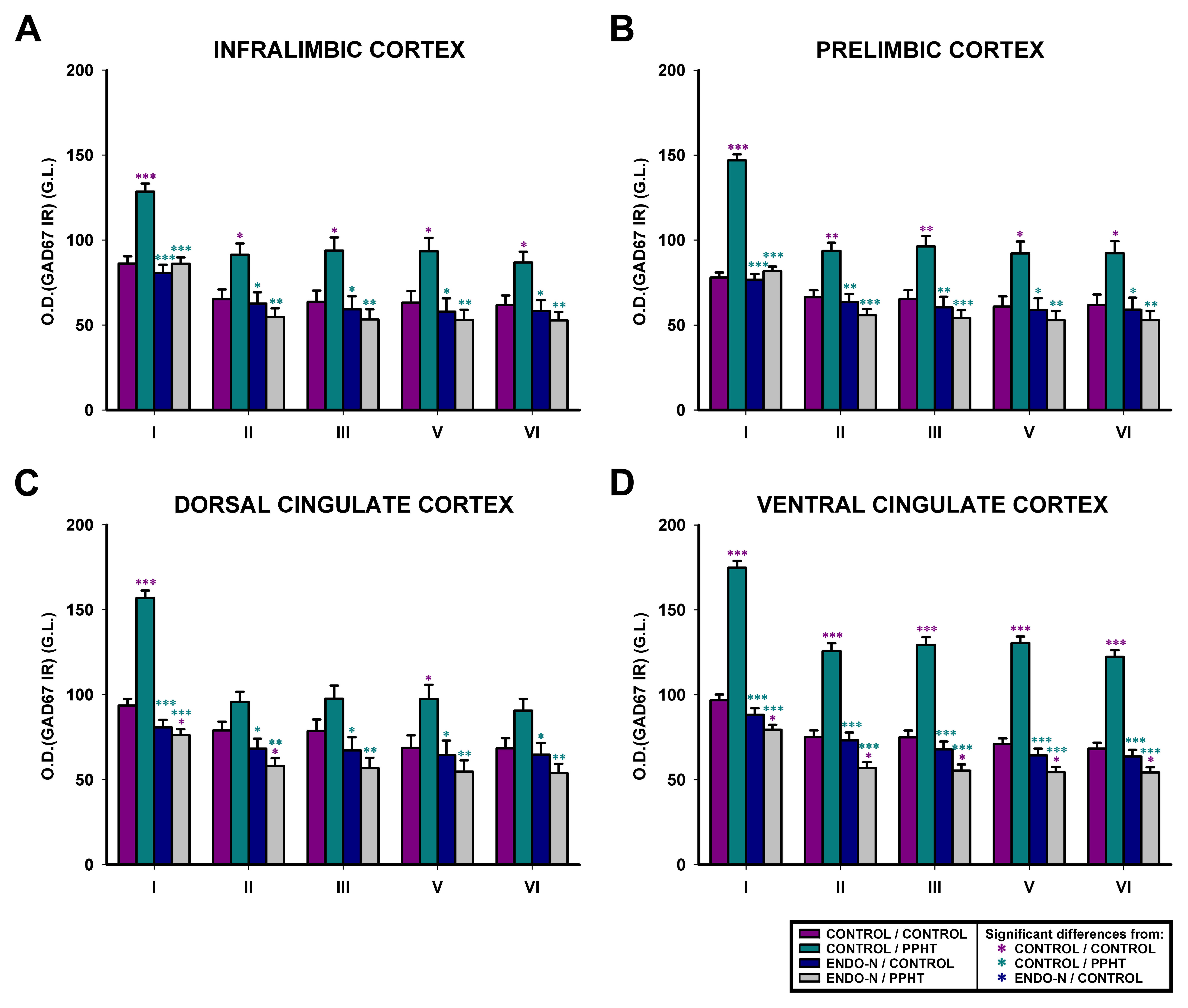

Supplement: Figure S6 — Graphs representing the changes in GAD67 neuropil expression after Endo-N and/or PPHT treatments. (A) Infralimbic cortex; (B) Prelimbic cortex; (C) Dorsal cingulate cortex; (D) Ventral cingulate cortex. Asterisks in bars indicate statistically significant differences between groups (see graph legend) after univariate repeated measures ANOVA followed by multiple pair-wise comparisons with Bonferroni's correction; p<0.05 (*), p<0.01 (**), p<0.001 (***). Roman numbers indicate cortical layers. (TIF) [file pone.0029516.s006.tif]
