# Supplementary material for: Polysialic Acid Is Required for Dopamine D2 Receptor-Mediated Plasticity Involving Inhibitory Circuits of the Rat Medial Prefrontal Cortex
Source: PLoS One. 2011 Dec 28;6(12):e29516. doi: 10.1371/journal.pone.0029516 (PMC3247286; doi:10.1371/journal.pone.0029516)
Supplement: Table S1 — Body weight analysis. Body weight data of all experimental animals in the day of surgery (day 0), before the onset of pharmacological treatment (day 7), in the middle of pharmacological treatment (day 10) and at the end of the experiment (day 14). Body weight differences across the different time points (day 0, 7, 10 and 14) were calculated ant then analyzed by one-way ANOVA tests (Inter-groups) followed, when appropriate, by multiple pair-wise comparisons with Bonferroni's correction. No statistically significant differences [n.s (p>0.05)] or statistically significant differences [p<0.05(*), p<0.01 (**), p<0.001 (***)] between groups. (DOC) [file pone.0029516.s007.doc]

| **Table S1. Body weight analysis** | | | | | | | | | | |  | |  | | |  | |  |  |  |
| --- | --- | --- | --- | --- | --- | --- | --- | --- | --- | --- | --- | --- | --- | --- | --- | --- | --- | --- | --- | --- |
|  |  | | **BODY WEIGHT (g)** | | | | | | | |  | **BODY WEIGHT DIFFERENCES (g)** | | | | | | | | |
| **RAT** | **GROUP** | | **Day 0**  **(d0)** | **Day 7**  **(d7)** | | | | **Day 10**  **(d10)** | **Day 14**  **(d14)** | |  | **d7-d0** | | | | **d10-d7** | | **d14-d10** | **d14-d7** | **d14-d0** |
| 1 | CONTOL / CONTROL | | 307 | 300 | | | | 309 | 319 | |  | -7 | | | | 9 | | 10 | 19 | 12 |
| 2 | CONTOL / CONTROL | | 301 | 272 | | | | 284 | 298 | |  | -29 | | | | 12 | | 14 | 26 | -3 |
| 3 | CONTOL / CONTROL | | 304 | 308 | | | | 307 | 312 | |  | 4 | | | | -1 | | 5 | 4 | 8 |
| 4 | CONTOL / CONTROL | | 303 | 295 | | | | 308 | 313 | |  | -8 | | | | 13 | | 5 | 18 | 10 |
| 5 | CONTOL / CONTROL | | 318 | 317 | | | | 327 | 331 | |  | -1 | | | | 10 | | 4 | 14 | 13 |
| 6 | CONTOL / CONTROL | | 304 | 305 | | | | 316 | 325 | |  | 1 | | | | 11 | | 9 | 20 | 21 |
| 7 | CONTROL / PPHT | | 286 | 284 | | | | 287 | 298 | |  | -2 | | | | 3 | | 11 | 14 | 12 |
| 8 | CONTROL / PPHT | | 294 | 292 | | | | 292 | 304 | |  | -2 | | | | 0 | | 12 | 12 | 10 |
| 9 | CONTROL / PPHT | | 298 | 286 | | | | 275 | 282 | |  | -12 | | | | -11 | | 7 | -4 | -16 |
| 10 | CONTROL / PPHT | | 289 | 291 | | | | 290 | 300 | |  | 2 | | | | -1 | | 10 | 9 | 11 |
| 11 | CONTROL / PPHT | | 310 | 325 | | | | 322 | 330 | |  | 15 | | | | -3 | | 8 | 5 | 20 |
| 12 | CONTROL / PPHT | | 295 | 291 | | | | 290 | 301 | |  | -4 | | | | -1 | | 11 | 10 | 6 |
| 13 | ENDO-N / CONTROL | | 285 | 273 | | | | 280 | 288 | |  | -12 | | | | 7 | | 8 | 15 | 3 |
| 14 | ENDO-N / CONTROL | | 294 | 279 | | | | 293 | 308 | |  | -15 | | | | 14 | | 15 | 29 | 14 |
| 15 | ENDO-N / CONTROL | | 302 | 294 | | | | 303 | 306 | |  | -8 | | | | 9 | | 3 | 12 | 4 |
| 16 | ENDO-N / CONTROL | | 290 | 275 | | | | 297 | 300 | |  | -15 | | | | 22 | | 3 | 25 | 10 |
| 17 | ENDO-N / CONTROL | | 293 | 290 | | | | 299 | 310 | |  | -3 | | | | 9 | | 11 | 20 | 17 |
| 18 | ENDO-N / CONTROL | | 302 | 294 | | | | 301 | 316 | |  | -8 | | | | 7 | | 15 | 22 | 14 |
| 19 | ENDO-N / PPHT | | 287 | 279 | | | | 274 | 279 | |  | -8 | | | | -5 | | 5 | 0 | -8 |
| 20 | ENDO-N / PPHT | | 292 | 291 | | | | 287 | 297 | |  | -1 | | | | -4 | | 10 | 6 | 5 |
| 21 | ENDO-N / PPHT | | 283 | 287 | | | | 282 | 287 | |  | 4 | | | | -5 | | 5 | 0 | 4 |
| 22 | ENDO-N / PPHT | | 277 | 282 | | | | 282 | 285 | |  | 5 | | | | 0 | | 3 | 3 | 8 |
| 23 | ENDO-N / PPHT | | 263 | 267 | | | | 257 | 259 | |  | 4 | | | | -10 | | 2 | -8 | -4 |
| 24 | ENDO-N / PPHT | | 326 | 329 | | | | 328 | 338 | |  | 3 | | | | -1 | | 10 | 9 | 12 |
|  |  | |  |  | | | |  |  | |  |  | | | |  | |  |  |  |
| **One way ANOVA tests** | | | | | | | | | | | | | | | | | | | | |
|  | |  | | |  | |  | | | | |  | | | | |  |  | **p-value** | |
| **d7-d0** | | Inter-groups | | |  | | | | | | | | | | | | | | 0,087 (n.s) | |
| **d10-d7** | | Inter-groups | | |  | | | | | | | | | | | | | | <0,001 ( ***) | |
|  | | Multiple pair wise comparisons | | |  | | | | | | | | | | | | | |  | |
|  | | | | | | CONTOL / CONTROL | | | | vs. | | | | | CONTROL / PPHT | | | | 0,005 ( **) | |
|  | | | | | |  | | | |  | | | | | ENDO-N / CONTROL | | | | 1,000 (n.s.) | |
|  | | | | | |  | | | |  | | | | | ENDO-N / PPHT | | | | 0,001 (**) | |
|  | | | | | | CONTROL / PPHT | | | | vs. | | | | | ENDO-N / CONTROL | | | | 0,001 (**) | |
|  | | | | | |  | | | |  | | | | | ENDO-N / PPHT | | | | 1,000 (n.s.) | |
|  | | | | | | ENDO-N / CONTROL | | | | vs. | | | | | ENDO-N / PPHT | | | | <0,001 (***) | |
| **d14-d10** | | Inter-groups | | | | | | | |  | | | |  | | | | | 0,322 (n.s) | |
| **d14-d7** | | Inter-groups | | | | | | | |  | | | |  | | | | | <0,001 (***) | |
|  | | Multiple pair wise comparisons | | | | | | | |  | | | |  | | | | |  | |
|  | | | | | | CONTOL / CONTROL | | | | vs. | | | | | CONTROL / PPHT | | | | 0,147 (n.s) | |
|  | | | | | |  | | | |  | | | | | ENDO-N / CONTROL | | | | 1,000 (n.s.) | |
|  | | | | | |  | | | |  | | | | | ENDO-N / PPHT | | | | 0,004 (**) | |
|  | | | | | | CONTROL / PPHT | | | | vs. | | | | | ENDO-N / CONTROL | | | | 0,017 (*) | |
|  | | | | | |  | | | |  | | | | | ENDO-N / PPHT | | | | 0,763 (n.s) | |
|  | | | | | | ENDO-N / CONTROL | | | | vs. | | | | | ENDO-N / PPHT | | | | <0,001 (***) | |
| **d14-d0** | | Inter-groups | | | | | | | |  | | | |  | | | | | 0,420 (n.s) | |
|  | |  | | | | | | | |  | | | |  | | | | |  | |
|  | | | | | | | | | | | | | | | | | | | | |
