# Supplementary material for: Polysialic Acid Is Required for Dopamine D2 Receptor-Mediated Plasticity Involving Inhibitory Circuits of the Rat Medial Prefrontal Cortex
Source: PLoS One. 2011 Dec 28;6(12):e29516. doi: 10.1371/journal.pone.0029516 (PMC3247286; doi:10.1371/journal.pone.0029516)
Supplement: Materials and Methods S1 — Supporting materials and methods. (DOC) [file pone.0029516.s008.doc]

**TITLE:** Polysialic Acid Is Required for Dopamine D2 Receptor-Mediated Plasticity Involving Inhibitory Circuits of the Rat Medial Prefrontal Cortex.

**SUPPLEMENTARY MATERIALS AND METHODS**

Endo-N stereotaxical injection and PPHT treatment

Twenty-four rats were deeply anaesthetized (5 mg/g xylazine and 0.5 ml/kg ketamine i.p.) and placed in a stereotaxic instrument (David Kopf Instruments, Tujunga, CA). A 10 μl Flexifil tapertip syringe (World Precision Instruments Inc.; Sarasota, FL) was then positioned in the secondary motor cortex, using the following coordinates from the atlas of Paxinos and Watson [1]: Bregma + 1,70 mm, Lateral ± 1,00 mm, Deep – 0,80 mm. The needle was left in position for 1 minute and then 1 μl of the enzime Endo-N (369 U/μl; AbCys, Paris, France) was injected over 1 minute period into one hemisphere in twelve rats. Control rats (n=12) received the same amount of the vehicle solution (NaCl 0.9% and glycerol, 1:1). After the injection was completed, the needle was left in place for 2 minutes to reduce reflux of the solution into the track of the injection needle and then withdrawn. The Endo-N is a phage enzyme that specifically cleaves alpha-2,8-linked sialic acid polymers with minimum chain length of 8. It diffuses rapidly throughout the brain and removes all detectable PSA within 1 day for 3-4 weeks [2]. For this reason, the injection was made on the secondary motor cortex, an area which is very close to the mPFC, which allowed us to deplete PSA without making any damage on the mPFC because of the needle. The effectiveness of Endo-N treatment was validated at the end of the experiment by means of a PSA-NCAM immunostaining. After recovery from anesthesia, rats were returned to their cages in the colony room, where they remained for one week prior to the start of pharmacological treatment.

Seven days after the intracranial injection, rats which had received Endo-N (n=12) were randomly separated in 2 groups (n=6) and administered intraperitoneally either the selective dopamine D2r agonist 2-(N-Phenethyl-N-propyl) amino-5-hydroxytetralin hydrochloride (PPHT, 1.5 mg/kg/day in 0.9% NaCl solution; Sigma-Aldrich) or saline (0.9% NaCl solution) once daily for 7 consecutive days [3]. The same procedure was followed for rats which had received intracranial vehicle injection (n=12). All animals were sacrificed 24 hours after the last intraperitoneal injection.

**Body weight analysis**

Body weight was measured in the day of surgery (day 0), before the onset of pharmacological treatment (day 7), in the middle of pharmacological treatment (day 10) and at the end of the experiment (day 14). Body weight differences across the different time points (day 0, 7, 10 and 14) were calculated. The Shapiro–Wilk's normality test and the Levene's test for homogeneity of variances showed that all the variables followed a normal distribution (p>0.05) and all samples had equal variances (p>0.05); therefore, data were analyzed by one-way ANOVA tests followed, when appropriate, by multiple pair-wise comparisons with Bonferroni’s correction (see **table S1** for further information).

Histological procedures

Rats were perfused transcardially under deep chloral hydrate anesthesia, first for 1 minute with NaCl 0,9 % and then for 30 minutes with 4% paraformaldehyde in sodium phosphate buffer 0.1 M, pH 7.4. Thirty minutes after perfusion, brains were extracted from the skull and their hemispheres were separated.

Both hemispheres of control rats (n=4) and the ipsilateral hemispheres respective to the side of injection of experimental rats (n=24) were cryoprotected with 30% sucrose in PB 0.1 M (4ºC) for 48 hours and then cut in coronal sections (50 μm) with a freezing-sliding microtome (Leica SM2000R; Leica, Nussloch, Germany). Slices were collected in 10 sequential subseries and stored at –20 ºC in a cryoprotective solution until used (30% glycerol, 30% ethylene glycol in PB 0,1M).

**Immunohistochemistry for conventional light microscopy**

Four subseries (50 μm-thick sections) from each treated animal were processed "free-floating" for immunohistochemistry using the avidin-biotin-peroxidase (ABC) method as follows. Sections were first incubated for 1 minute in an antigen unmasking solution (0.01 M citrate buffer, pH 6) at 100ºC. After cooling down the sections to room temperature, they were incubated with 3% H_2_O_2_ in phosphate buffered saline (PBS) for 10 minutes to block endogenous peroxidase activity. After this, sections were treated for 1 hour with 10% normal donkey serum (NDS) (Jackson ImmunoResearch Laboratories, West Grove, PA) in PBS with 0.2% Triton-X100 (Sigma-Aldrich, St. Louis, MO) and were incubated for 24 or 48 hours (see **table 1**) at 4ºC in primary antibody (anti-PSA-NCAM, anti-SYN, anti-GAD67 or anti-NCAM antibody). After washing, sections were incubated for 2 hours (room temperature) with the proper biotinilated secondary antibody (anti-mouse IgM or anti-mouse IgG), followed by an avidin-biotin-peroxidase complex (ABC; Vector Laboratories, Peterborough, UK) for 1 hour in PBS. Color development was achieved by incubating with 3,3’- diaminobenzidine tetrahydrochloride (DAB; Sigma-Aldrich) and 0.033% H_2_O_2_ for 4 minutes. PBS containing 0.2% Triton-X100 and 3% NDS was used for primary and secondary antibodies dilution. Please, see **table 1** for further information about antibodies.

All the studied sections passed through all procedures simultaneously in order to minimize any difference from immunohistochemical staining itself. To avoid any bias in the analysis, all slides were coded prior to analysis and the codes were not broken until the experiment was finished.

Quantification of neuropil immunoreactivity

From each immunostaining (PSA-NCAM, SYN, GAD67 and NCAM), three sections per animal were selected randomly from the following coordinate intervals (infralimbic cortex: Bregma 2.70 to 2.20 mm; prelimbic cortex: Bregma 3.70 to 2.20 mm; cingulate cortex: Bregma 1.70 to -0.26 mm. [1]). Sections were examined with an Olympus CX41 microscope under bright-field illumination, homogeneously lighted and digitalized using a CCD camera. Photographs to the different areas and layers were taken at 20X magnification. A Nissl staining in alternate series of sections was used for determining layer and area boundaries within medial prefrontal cortex regions, based on cytoarchitectural differences across these layers and areas (see **fig. S3**). Grey levels were converted to optical densities (OD) using Image J software (NIH). Means were determined for each experimental group and data were subjected to one-way repeated-measures ANOVA using the SPSS software package (version 17).

Region (infralimbic, prelimbic, dorsal cingulate and ventral cingulate cortices) and layer (I, II, III, V, VI) were considered as within-subjects variables (repeated measures variables) and treatment (Endo-N/PPHT, Endo-N/Control Control/PPHT, Control/Control), as between-subjects variable. The sphericity assumption in repeated measures variables and the homogeneity of variances in between-subjects variable were first assessed by means of the Mauchly’s test of sphericity and the Levene’s test for homogeneity of variances.

Estimation of the total number of neurons expressing PSA-NCAM, CB, CR or PV

Neuronal somata expressing PSA-NCAM, CB, CR or PV covering 100% of the sample area were counted, that is, within each 50 µm-thick section, all labeled cells found in the selected region (infralimbic, prelimbic, dorsal cingulate and ventral cingulate cortices) and layer (I, II, III, V and VI). The fractionator sampling scheme refers to the methodology of examining one out of every ten brain sections. Thus, our modification of the optical dissector combined with a 1:10 fractionator sampling is truly a modification of the optical fractionator method. One from 10 systematic-random series of sections covering the whole rostral to caudal extension of mPFC was viewed on an Olympus CX41 microscope. Cell somata were identified and counted with a 40X objective. Cells appearing in the upper focal plane were omitted to prevent counting cell caps.

Since Endo-N intracranial injection produced an absolute absence of PSA-NCAM immunoreactive neurons in the mPFC **(fig. 4)**, only non-Endo-N injected animals were analyzed. Means were determined for each experimental group (Control/PPHT, Control/Control) within each region and layer from the mPFC area and data were then subjected to unpaired Student’s t-test statistical analysis.

**Immunohistochemistry for confocal microscopy**

In general, tissue was processed "free-floating" for immunohistochemistry as described above but omitting the endogenous peroxidase block. Sections were incubated for 24 or 48 hours at 4ºC with proper primary antibody cocktails (see table 1 and materials and methods). After been washed, sections were incubated at room temperature and light-protected with proper fluorescent secondary antibody cocktails (see **table 1**) for 2 hours.

Goat anti-mouse IgG subclass 1 specific was used for the detection of anti-CaMKII-α primary antibody when other primary antibody generated in mouse was also being used. Because this secondary antibody was conjugated with biotin, the Avidin, NeutrAvidin®, Texas Red® conjugate (1:200, Molecular Probes) or streptavidin, Alexa Fluor ® 488 conjugate were used before to detect biotin.

All sections processed for fluorescent immunohistochemistry were mounted on slides and coverslipped using DakoCytomation fluorescent mounting medium (Dako North America Inc., Carpinteria, CA).

**Specificity of primary antibodies**

The specificity of all the primary antibodies employed in our study has been tested and confirmed by their commercial providers using western blot analysis of rat brain homogenates. In addition, the commercial providers and/or previous studies have tested them by immunohistochemistry in paraformaldehyde fixed rat brains (see references o**n table 1)**. All these antibodies showed a regional and cellular immunolabeling distribution comparable to that of their respective antigens with the same or other equally selective antisera. In order to confirm that some of the immunostaining was not produced by the secondary antibodies or by the immunohistochemical protocol, we have omitted primary antibodies or we have substituted them by normal donkey serum. These controls resulted in a complete absence of immunostaining in every case.

**REFERENCES**

1. Paxinos G, Watson C (1986) The rat brain in stereotaxic coordinates. Academic Press, London.
2. Troy FA, Hallenbeck PC, McCoy RD, Vimr ER (1987) Detection of polysialosyl-containing glycoproteins in brain using prokaryotic-derived probes. Methods Enzymol 138: 169-185.
3. Castillo-Gomez E, Gomez-Climent MA, Varea E, Guirado R, Blasco-Ibanez JM et al (2008) Dopamine acting through D2 receptors modulates the expression of PSA-NCAM, a molecule related to neuronal structural plasticity, in the medial prefrontal cortex of adult rats. Exp Neurol 214: 97-111.
